# Strategizing Drug Therapies in Pulmonary Hypertension for Improved Outcomes

**DOI:** 10.3390/ph15101242

**Published:** 2022-10-10

**Authors:** Taylor Beckmann, Patrisha Shelley, Darshan Patel, Mounica Vorla, Dinesh K. Kalra

**Affiliations:** 1 Department of Medicine, University of Louisville School of Medicine, Louisville, KY 40202, USA; 2 Division of Pulmonary and Critical Care Medicine, Department of Medicine, University of Louisville School of Medicine, Louisville, KY 40202, USA; 3 Division of Cardiology, Department of Medicine, University of Louisville School of Medicine, Louisville, KY 40202, USA

**Keywords:** pulmonary hypertension, drug targets, mechanisms of drug action, therapeutic approaches, basic research, translational research, reverse translational research

## Abstract

Pulmonary hypertension (PH) is characterized by a resting mean pulmonary artery pressure (PAP) of 20 mmHg or more and is a disease of multiple etiologies. Of the various types of PH, pulmonary arterial hypertension (PAH) is characterized by elevated resistance in the pulmonary arterial tree. It is a rare but deadly disease characterized by vascular remodeling of the distal pulmonary arteries. This paper focuses on PAH diagnosis and management including current and future treatment options. Over the last 15 years, our understanding of this progressive disease has expanded from the concept of vasoconstrictive/vasodilatory mismatch in the pulmonary arterioles to now a better appreciation of the role of genetic determinants, numerous cell signaling pathways, cell proliferation and apoptosis, fibrosis, thrombosis, and metabolic abnormalities. While knowledge of its pathophysiology has expanded, the majority of the treatments available today still modulate the same three vasodilatory pathways that have been targeted for over 30 years (endothelin, nitric oxide, and prostacyclin). While modifying these pathways may help improve symptoms and quality of life, none of these directly modify the underlying disease pathogenesis. However, there are now studies ongoing with new drugs that can prevent or reverse these underlying causes of PAH. This review discusses the evidence base for the current treatment algorithms for PAH, as well as discusses novel therapies in development.

## 1. Introduction

Pulmonary hypertension (PH) is a severe, life-threatening disease with adverse effects on the cardiopulmonary system leading to high levels of morbidity and mortality. Over time, PH leads to pulmonary vascular remodeling and increased pulmonary vascular resistance which can cause right ventricular failure and the systemic consequences of right heart failure, eventually culminating in death. Elevated mean pulmonary artery pressure (mPAP) > 20 mmHg measured on resting right-heart catheterization is the defining characteristic in all categories of PH [1]. The most common etiology of PH originates from left-heart disease (LHD) with the next most common one being parenchymal lung disease. A rarer variant of PH is pulmonary arterial hypertension (PAH), a disease where the underlying pathophysiology originates in the pulmonary artery (PA) vasculature. A combination of inciting factors (drugs, inflammation, infection, etc.) and genetics results in arterial intimal proliferation as one of the first steps in disease initiation. This is followed by concentric or eccentric laminar sclerosis, resulting in medial hypertrophy and adventitial proliferation. Plexiform lesions consisting of accumulated endothelial and inflammatory cells within occluded arteries may be seen in advanced disease. Such lesions eventually lead to progressive vascular fibrosis and elevations in PVR [2]. While the initial histopathological abnormalities originate in the pulmonary arterioles, disease mortality is largely determined by RV dysfunction in these patients [2].

The hemodynamic effects of elevated pulmonary artery pressure (PAP) are felt systemically with consequences of right ventricle (RV) failure, hepatic congestion, chronic kidney disease, hypoxemia, and impaired neurohormonal signaling. These consequences manifest as globally impaired cardiovascular fitness, function, and eventually death. The presenting symptoms are nonspecific with the most common being dyspnea on exertion, fatigue, and lethargy. Other symptoms include exertional chest pain, weight gain, fluid retention, exertional syncope, and anorexia [3]. The signs of PH include jugular venous pressure elevation, edema, ascites, and hepatomegaly. These symptoms may get attributed to other diseases, leading to delayed diagnosis. An estimated 20% of PH is symptomatic for 2 years before the correct diagnosis is made [4].

Despite years of focused research and improvements in medical therapies, the mortality of group 1 PAH remains high [3]. The 1, 2, and 3 year mortality rates for all PAH patients based on combined registry data are 8%, 16%, and 21%. These numbers include patients in all risk stratification categories, patients naïve to therapy, and patients currently on therapy [5]. The currently approved medications for PAH primarily focus on symptomatic treatment through one of three vasodilatory pathways involved in the pathogenesis of the disease. There are currently no approved medications that modify the disease process. These three pathways remain the only treatment focus since the 1990s despite the discovery of new molecular pathways involved in PAH. The current guidelines and treatment algorithms are the focus of this paper, along with novel therapy targets under development.

## 2. Physiology

The pulmonary vascular circuit consists of a network of arteries, capillaries, and veins that are responsible for the gas exchange of the entire circulatory system. In contrast to the remainder of the circulatory system that functions as a series of circuits, the pulmonary vasculature functions as a parallel circuit to permit high flow with low pressure and resistance [3,6]. These parallel circuits continuously branch and narrow into smaller diameters to maximize the surface area of capillary beds and optimize gas exchange. The small-caliber arterioles measure less than 100 µm in diameter, the muscular arteries measure 100–500 µm, and the large elastic arteries of the pulmonary tree may be over 500 µm [7,8]. The large elastic arteries are less muscular and more distensible at the low transmural pressures they typically receive. The medium-caliber muscular arteries decrease in elastic properties compared to larger vessels and instead gain more smooth muscle, specifically in the tunica media layer [7]. The smooth muscle controls the blood pressure of the pulmonary circuit via vasoconstriction and dilation. The outer layer of the adventitia is composed of progenitor cells, vasa-vasorum, and fibroblasts [6,7,8]. The small diameter arterioles comprise similar but thinner layers than the muscular arteries. These vessels have fewer medial smooth muscle cells to reduce the driving pressure in the downstream capillaries. The medium and small muscular PAs are the main site of arterial dysfunction in Group 1 PH or PAH, although all types will have some level of vascular remodeling in these vessels. New evidence supports the involvement of the proximal, larger PAs in the adverse vascular remodeling and right ventriculo-arterial coupling. Vascular stiffening in the pulmonary arterial bed precedes the development of PH [9]. Increased flow in the stiffer, proximal larger PA increases pressure and pulsatility (shear and barotrauma) in the distal, highly compliant, smaller PAs, eventually leading to their muscularization and remodeling [10]. This results in higher mean arterial pressures, which then cause extensive vessel wall remodeling, as well as stiffening of the large PAs [11]. Thus, these changes result in a positive feedback cycle of pathologic vascular remodeling. Vascular stiffening also promotes local remodeling through alterations in gene expression and cellular behavior in response to the local mechanico-biological feedback [12]. This pathologic remodeling and fibrosis lead to higher pressures; consequently, the main PA dilates. The increased afterload leads to increased RV pressures and eventually RV hypertrophy over time [6,7]. In the later stages of PAH, the dilated and weak RV eventually cannot pump blood forward effectively, leading to right-heart failure.

### Classification

The single hemodynamic criterion needed to make the diagnosis of PH is mPAP > 20 mmHg which is best measured on right-heart catheterization. This is a change from the original mPAP > 25 mmHg established in the 1970s by the WHO. A revised definition of PH was proposed in 2018 by a task force at the sixth World Symposium on Pulmonary Hypertension [1]. These new criteria are aimed at increasing the sensitivity of diagnosing PH as recent data suggest that even an mPAP > 20 mmHg carries an increased risk of mortality, although these data may not adjust for confounders such as increased age, BMI, and comorbidities of LHD or lung disease [1]. Another reason for the change may be that many PAH cases still go undiagnosed for two or more years from the onset of symptoms to the time of diagnosis. The change in definition comes with some controversy as the majority of the current data on the safety and efficacy of pharmacologic therapy have been collected on patients with a mPAP > 25 mm Hg and not in the population with mPAP > 20 and mPAP < 25, which was previously considered a borderline elevation [1]. Current and future studies will now need to include this population to ensure that this subgroup also shows the same degree of benefit with guideline-directed therapy.

**Figure 1:**
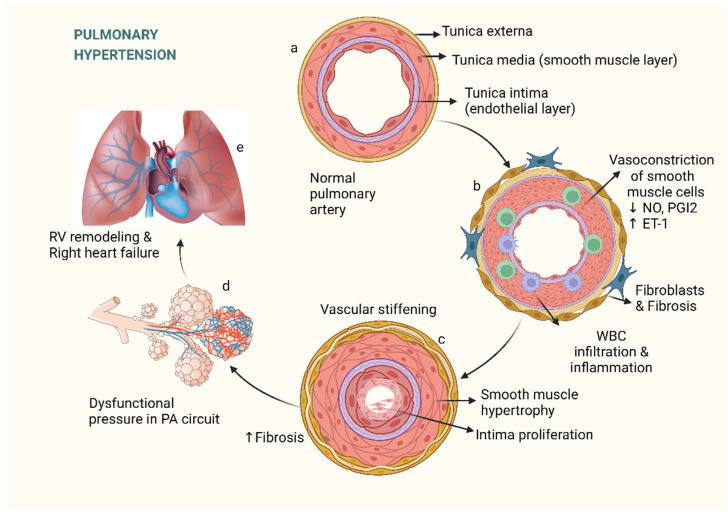
(**a**) Cross-section of normal pulmonary artery with tunica intima, media, and externa. (**b**) Dysregulated cellular metabolic events lead to decreased (↓) vasodilator production (nitric oxide [NO] and prostacyclin [PGI2]) and overproduction (↑) of vasoconstrictors (endothelin-1 [ET1]), leading to increased and sustained vasoconstriction via smooth muscle contraction. Pulmonary vascular remodeling and inflammation ensues (**c**) Sustained vasoconstriction leads to smooth muscle hypertrophy. Proinflammatory cytokines, serotonin, bone morphogenetic proteins (BMPs), and members of the transforming growth factor (TGF-β) family lead to proliferation of the extracellular matrix and fibroblasts, causing fibrosis and resulting in vascular stiffening and creation of plexiform lesions (**d**) Dysfunctional pressure regulation in the pulmonary circuit leads to elevated mPAP and abnormal RV-PA coupling. (**e**) PAH eventually culminates in right-heart failure.

A World Health Organization (WHO) classification system of PH exists that is based on the underlying mechanisms of disease; this classifies the disease into five separate groups, as shown in Figure 1. Group 1 represents PAH, Group 2 represents LHD, Group 3 represents intraparenchymal lung disease, Group 4 represents chronic thromboembolic pulmonary hypertension (CTEPH), and Group 5 includes a heterogeneous mix of etiologies.

Another scheme of classification groups PH into pre- and post-capillary dysfunction on the basis of whether the hemodynamic abnormality is in the pulmonary venous (postcapillary) or the arterial (precapillary) bed. The distinction is made on the basis of pulmonary capillary wedge pressure (PCWP) measured during RHC, which transmits left-ventricular end-diastolic pressure in the absence of left-sided valvular disease. In other words, a PCWP > 15 mmHg represents post capillary dysfunction or venous PH while a PCWP < 15 mmHg represents pre-capillary PH [8,13]. These classifications exist not only for understanding the etiologies but also for guiding treatment. Treatment for Group 2 or post-capillary dysfunction is geared toward correcting LHD. The majority of PH clinicians will see is Group 2 or post-capillary PH. Pulmonary vascular resistance (PVR) (typically measured in Wood units) is another important hemodynamic measurement that gives insight into the nature and severity of PH. PVR measured on RHC as (mPAP (left-atrial pressure or PCWP)/CO)) represents the ratio of transpulmonary pressure to pulmonary artery blood flow [13,14]. PVR can help delineate those post-capillary PH patients who also have a significant pre-capillary component (i.e., combined pre- and post-capillary PH); these patients will have an elevated mPAP (>20 mm Hg) with a high PVR (>2 WU) and a high PAWP (>15 mm Hg).

Although mPAP provides prognostic information across all etiologies of PH, there are a few reversible physiological states of elevated mPAP such as anemia, pregnancy, and high-performance athletes. Thus, PVR is used as a hemodynamic surrogate of pulmonary vascular disease to help increase specificity in diagnosing PH [3,15]. Evaluation of PVR in combination with mPAP more accurately tracks the progression of disease course.

The early hemodynamic change evident in PAH is an elevation of PAPs due to muscularization of medium to small-sized arteries in combination with other forms of vascular remodeling as seen in Figure 1. Unfortunately, using mPAP as a marker of disease progression has some downside since mPAP starts increasing early on in the disease. The mPAP will typically peak just prior to the development of right-ventricular heart failure, which may be when patients start to develop severe symptoms [3,6]. Since PAP is mainly determined by the contractility of the RV, RV failure may lower previously elevated mPAPs and, thus, mask progression of the disease. PVR represents a better hemodynamic metric for staging PH as it is inversely related to cardiac output (CO) and continues to increase, even in the late stages of disease progression.

## 3. Group 1 PH: Pulmonary Arterial Hypertension

Pulmonary arterial hypertension is a rare form of PH that affects 15 to 50 per million people and is diagnosed at a mean age of 53 years. There is a female dominance (female-to-male ratio of ~ 3:1). PAH is characterized hemodynamically by mPAP > 20 mmHg, PVR > 2 Wood units (recently updated per the 2022 ESC guidelines), and PCWP < 15 mmHg. PAH can be idiopathic (IPAH), heritable, or associated with connective tissue diseases (CTD) such as systemic sclerosis, congenital heart diseases, human immunodeficiency virus, schistosomiasis infection, portal hypertension, or exposure to toxins/drugs [13]. In all these subgroups of PAH, there is an imbalance in the homeostatic levels of endothelial vasoactive factors such as nitric oxide (NO), prostacyclin, and endothelin (ET). Even with the advancement in treatment options over the last 30 years, the 7 year mortality remains at approximately 49% [13,16].

### 3.1. Genetics

In recent years, genetic pathways in the pathogenesis of PAH have been an area of heavy emphasis. Global registries including the REVEAL registry place the incidence of heritable PAH at 5% or less. The bone morphogenetic protein receptor 2 (BMPR2) gene has been identified as a major player in the pathogenesis of both heritable and nonheritable forms of PAH. Mutations in BMPR2 are found in up to 80% of heritable PAH cases and between 11% and 40% of IPAH cases [13,17,18]. The BMPR2 receptor is a serotonin/threonine kinase receptor in the transforming growth factor-B (TGF-β) superfamily that activates multiple intracellular signaling cascades. Mutations in the BMPR2 gene lead to unopposed TGF-β signaling [8]. The majority of BMPR2 expression is found in pulmonary endothelial cells, vascular smooth muscle cells (SMC), and macrophages. BMPR2 signaling plays a major role in regulating vascular cell apoptosis in the pulmonary arterial wall. A reduction in signaling leads to endothelial inflammation and loss of regulated cell apoptosis, which causes maladaptive vascular remodeling. Even though a higher frequency of BMPR2 mutations is seen in those with PAH, while these mutations increase susceptibility, environmental stresses are still required for the disease to manifest [8,17]. Reduced BMPR2 signaling has also been seen in PAH patients without BMPR2 receptor mutations. These findings have made this signaling pathway a target of interest for pharmacologic therapy.

### 3.2. Pathogenesis

While the exact mechanism that initiates the pathological changes in PAH is unknown, a combination of known risk factors and genetic predisposition is likely operational. An initial insult to the distal pulmonary arterial tree likely starts a cascade of maladaptive inflammatory responses in predisposed individuals. This cascade results in vasoconstriction and inflammation with a population of apoptosis-resistant SMCs that promote fibrosis [Figure 1]. Elevated vascular tone and vascular remodeling involve abnormalities in several cell types including endothelial cells, SMCs, and fibroblasts. In the adventitia, there is overproduction of extracellular matrix leading to fibrosis and loss of elastance. These complex yet interrelated vascular abnormalities come together to form a plexiform lesion, the hallmark of group 1 PH [13]. Additionally, growth factors such as vascular endothelial growth factor (VEGF), platelet-derived growth factor (PDGF), insulin-like growth factor-1 (IGF-1), and epidermal growth factor (EGF), play an important role in the maladaptive vascular remodeling responsible for the rise in the PVR in PAH (Figure 1) [6,8,18]. These growth factors have been found in elevated levels in the sera and bronchoalveolar lavage samples of PAH patients. This maladaptive remodeling leads to vascular obstruction and reduced compliance, increasing RV afterload and impairing right-ventricular–pulmonary artery (RV–PA) coupling. This increase in RV afterload leads to RV dilation and eventually RV failure. RV functional and structural alterations are the main determinants of prognosis and survival, and they contribute to the symptoms of edema, systemic congestion, ascites, anorexia, syncope, and fatigue that patients experience.

Below, we highlight important cellular pathways that have been historical targets of drug therapy.

#### 3.2.1. Endothelin

Endothelin (ET) is a potent vasoconstrictor produced by endothelial cells. Excessive vasoconstriction in PAH has been linked to upregulated ET. Two distinct receptors, ET-A and ET-B, produce the vasoconstrictive effects of ET; these receptors are targets of the endothelin receptor antagonist (ERA) class of medications [17,19,20]. Activation of ET-A receptors causes proliferation of vascular smooth-muscle cells and sustained vasoconstriction. ET-B receptors mediate pulmonary ET clearance and induce the production of NO and prostacyclin by endothelial cells that counterbalance the deleterious effects of ET.

#### 3.2.2. Nitric Oxide

Nitric oxide (NO), produced by nitric oxide synthase from the precursor L-arginine, mediates powerful vasodilatory effects on SMCs. NO activates soluble guanylate cyclase, leading to increased cyclic guanosine monophosphate (cGMP) levels. cGMP acts directly on smooth muscle cells resulting in vasodilatation; it also reduces smooth muscle proliferation and platelet aggregation and thrombosis. cGMP is typically metabolized through phosphodiesterase type 5 (PDE5). In PAH, endothelial dysfunction triggers reduced levels of endothelial NO synthase and increased levels of PDE5, leading to lower NO production and increased cGMP degradation [17,21].

#### 3.2.3. Prostacyclin

Prostacyclin (also called prostaglandin I2) is a member of the endogenous prostanoid family and is principally produced by endothelial cells. The enzymes cyclo-oxygenase (COX) and prostacyclin synthase convert arachidonic acid (AA) into prostacyclin in a multistep process. This reaction takes place preferentially with COX-1, which is also responsible for the synthesis of thromboxane, a potent clotting factor from AA. Since these end products seemingly work in opposition, the determining factor in what product will be made is the presence of the second enzyme, prostacyclin synthase or thromboxane synthase [17,22,23]. Different cell types have different concentrations of each; endothelial cells express more prostacyclin synthase while platelets express mostly thromboxane synthase. Prostacyclin exerts its effects by binding to the prostacyclin receptors and increasing cyclic adenosine monophosphate (cAMP) levels leading to vasodilation, inhibition of platelet aggregation, improvement of endothelial dysfunction, increase in right heart inotropy, and antiproliferative activities. Patients with PAH have a marked reduction in prostacyclin synthase, lower plasma levels of prostacyclin metabolites, and decreased expression of prostacyclin receptors in the lungs.

### 3.3. Diagnosing Pulmonary Arterial Hypertension

#### 3.3.1. Clinical Assessment

As mentioned, the presenting symptoms for PAH can be vague, nonspecific, and, in the later stages, related to RV dysfunction. The most common symptom is dyspnea on exertion, which was reported in over 80% of patients in the REVEAL registry [4,13]. Other common symptoms include shortness of breath, fatigue, weakness, angina, and syncope. Less common symptoms include a dry cough and exercise-induced nausea. As the disease progresses, symptoms will occur at rest. Due to the nonspecific nature of these symptoms, PAH commonly goes undiagnosed until an average of 2 years after symptom onset [4].

**Figure 2:**
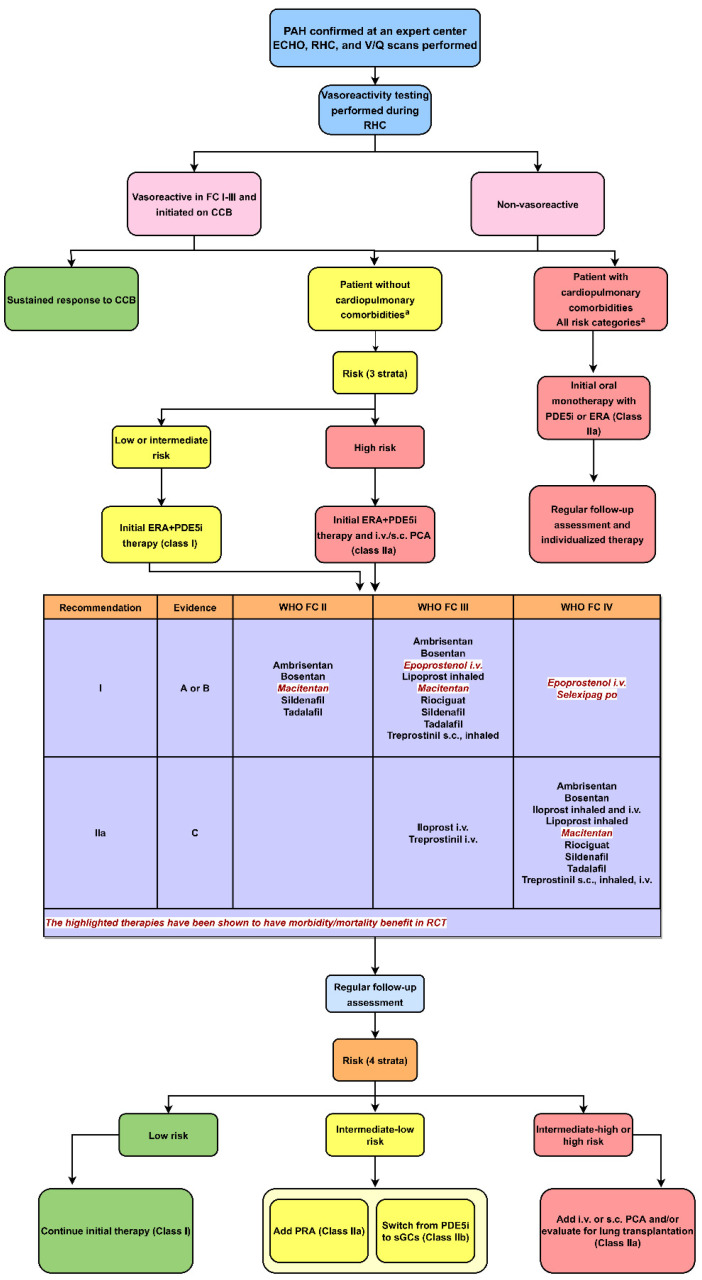
Treatment algorithm for PAH based on current guidelines.

On exam, jugular venous distention, right-sided murmurs, hepatomegaly, and peripheral edema may all be present. These signs may be missed unless carefully looked for, and this likely accounts for the delayed diagnosis in patients with PAH. Some symptoms and signs may be incorrectly attributed to common conditions such as obesity, deconditioning, asthma, chronic pulmonary disease, or even anxiety. Patients presenting with new unexplained shortness of breath should have a stepwise cardiopulmonary workup of their symptoms. Common initial tests include a 6 min walk test with oximetry, chest X-ray, pulmonary function tests, routine blood tests (including hematocrit and thyroid function tests), and transthoracic echocardiography (TTE) (Figure 2). TTE is one of the most informative noninvasive imaging cardiac tests in the initial workup of PAH [13,14,24]. A PA systolic pressure (estimated by applying the Bernoulli equation, PASP = right atrial pressure + 4 × (maximal TR velocity)^2^] of greater than 35 mmHg or an mPAP greater than 20 mmHg in the absence of LHD should prompt a more detailed workup for PAH once pulmonary diseases have been ruled out (the latter is called group 3 PH, and typically results in PASP in the range of 40–60 mmHg). Depending on the clinician’s level of suspicion for PH, referral to an expert PAH center should be considered, where a right-heart catheterization may be pursued to obtain accurate invasive measurements of PA, PCWP, CO, and PVR [14]. If these are abnormal, vasoreactivity testing should be performed during the initial RHC. Numerous nuances in the diagnostic pathway for PAH exist but are not the focus of this paper. Pulmonary ventilation and perfusion scanning (V/Q scan) should also be performed to investigate mismatched perfusion defects that would indicate group 4 PH CTEPH. There are emerging data for other imaging modalities such as cardiovascular MRI to get a detailed look at RV and LV structure and function, rule out shunts, look at flow patterns in the PA, and assess for myocardial tissue characteristics (T1 mapping) and LGE (late gadolinium enhancement), which add further information to disease staging and prognosis.

#### 3.3.2. Staging PAH

A thorough and accurate clinical assessment is of extreme importance in managing PAH as it provides information for determining disease severity, improvement, deterioration, or stability. The WHO functional class (FC) has been a long-established predictor of survival even given the interobserver variability. Monitoring for changes in the FC alerts providers to disease progression and should drive intensification of the treatment regimen. The FC is driven by the patient’s reported symptoms and activity limitations. The 2022 ESC guidelines recommended a four-strata classification system including the WHO-FC class to help drive more accurate therapy and decision-making [5]. FC ranges in severity from I to IV, which can also be stratified as low, intermediate–low, intermediate–high, and high risk (Figure 2). Using FC, an estimate of exercise capacity or the 6 min walk distance (6MWD), and a proxy for RV function (such as pro-BNP [B-type natriuretic peptide] or TTE), patients can be grouped into one of the four strata categories, and a 1-year mortality estimate can be calculated [7,19]. Using this staging system, low-risk (FC-I), intermediate–low-risk (FC-II), intermediate–high-risk (FC-III), and high-risk (FC-IV) patients will experience a 0–3%, 2–7%, 9–19%, >20% mortality risk at 1 year [3]. The comprehensive risk assessment in pulmonary arterial hypertension remains a three-strata model and includes imaging modalities such as echocardiography, cardiac MRI, and hemodynamics found on RHC. The comprehensive assessment is recommended at diagnosis, while the four-strata model is recommended at follow-up as a basic risk stratification tool, but additional variables should be considered as needed [5].

More sophisticated and evidence-based risk calculators have been developed to help guide care and allow for more accurate mortality predictions. These include the US Registry to Evaluate Early and Long-term PAH Disease Management (REVEAL) risk equation and the revised REVEAL 2.0 [25]. The French Pulmonary Hypertension Network registry risk equation and the 2022 European Society of Cardiology/ European Respiratory Society guidelines of PH are alternative options to the REVEAL 2.0. These scoring systems have all been validated in separate datasets and are routinely employed in clinical drug trials to monitor disease progression and uptitration of pharmacologic therapy [13,14,26,27].

### 3.4. Current Medical Therapies for PAH

#### 3.4.1. Vasoreactivity Testing and Calcium Channel Blockers

The purpose of vasoreactivity testing in PAH is to identify acute vasoresponders who may be candidates for treatment with high-dose calcium channel blockers (CCBs). Pulmonary vasoreactivity testing is only recommended in patients with idiopathic PAH, heritable PAH, or drugs/toxins associated PAH [28]. This should be performed during the RHC so that the hemodynamic response to vasodilators can be evaluated at the time of confirmation of the diagnosis of PAH. Per the 2022 ESC guidelines, inhaled nitric oxide or inhaled iloprost are the recommended test compounds for vasoreactivity testing (Figure 2). There is similar evidence for intravenous (IV) epoprostenol; however, due to incremental dose increases and repetitive measurements, testing takes much longer and is, therefore, less feasible. Intravenous adenosine is not currently recommended due to the frequent occurrence of side-effects [28]. A positive test is a drop in the mPAP by at least 10 mm Hg to a final value below 40 mm Hg and a stable or increased CO. Such patients are termed as having a positive vasoreactivity test. They more commonly have either IPAH or anorexigen-induced PAH as the etiology and have a favorable prognosis compared to those who do not show a vasoreactive response to this vasodilator challenge. These patients show a good initial treatment response to calcium channel blockers and may even experience a mortality benefit if they demonstrate a sustained response [14,15]. However, only a small portion of acute vasodilator responders have shown long-term benefits to calcium channel blocker therapy; hence, continued hemodynamic monitoring on therapy is needed. Oral amlodipine, diltiazem, and long-acting nifedipine are the commonly used therapies. The choice among these three is typically based on the patient’s resting heart rate, as tachycardia favors the use of diltiazem, while bradycardia favors the use of nifedipine or amlodipine. The effective daily doses that have been used in PAH are relatively high compared to the usual dosages used in systemic hypertension. We often use 120–240 mg/day for nifedipine, 240–480 mg for diltiazem, and 20–30 mg for amlodipine. Starting at a lower dose and titrating up is advisable to avoid hypotension. An adequate response means a stable FC I–II patient who achieves near-normal hemodynamics. However, if such a response is not initially achieved or sustained over time, then other PAH therapy should be initiated [29].

#### 3.4.2. Prostacyclin Pathway

Prostacyclin therapies were introduced in the 1990s and immediately increased PAH survival rates from 2 years to about 5–7 years [17,21,22]. These endogenous prostanoids act as potent vasodilators by binding to the prostaglandin I (IP) receptor on SMCs, fibroblasts, endothelial cells, and platelets. This increases cAMP expression to mediate their vasodilatory effects. The agents used today are epoprostenol, iloprost, treprostinil, and selexipag.

Epoprostenol was the first available prostacyclin analog, and it is administered parenterally as a continuous infusion (half-life of 3–5 min) with a permanent tunneled catheter. The need for continuous venous access carries the potential risk of catheter-associated bloodstream infections. Other frequent side-effects of IV epoprostenol include headache, jaw pain, diarrhea, nausea, flushing, erythroderma, anxiety, and musculoskeletal pain, although some of these improve with time and by going up slowly on the dose [17,21]. Epoprostenol historically had to be kept cool with an ice pack as it is unstable at room temperature. A more basic (pH) formulation approved in 2012 requires no ice pack [22]. More stable prostacyclin analogs have been created to overcome these issues.

Treprostinil has similar pharmacokinetics to epoprostenol but is more stable at room temperature and has a half-life of 4 h [13]. Treprostinil can be administered subcutaneously, intravenously, by inhalation, or orally. While parenteral treprostinil shares the same side-effects as epoprostenol, the subcutaneous (SQ) formula reduces the risk of bloodstream infections, but 85% of patients still experience some injection site pain, especially when first administered [30]. A fully implantable pump has also been developed and improves patient quality of life. SQ treprostinil may take 6 months to reach a stable dose and often requires gradual dose increases [17,21,30]. Inhaled treprostinil minimizes the typical side-effects of IV prostacyclin due to reduced systemic absorption [31]. It also lacks catheter-related risks. The most common side-effects of the inhaled route of delivery are a dry cough and headache. Treprostinil diethanolamine is a salt form of treprostinil designed for twice-daily oral dosing [31]. Iloprost is another stable synthetic prostanoid found in IV (not approved in the US or Europe), inhaled, and oral forms with a half-life of 20 min. Selexipag is an oral non-prostanoid selective agonist for IP receptors that has more specificity to the IP receptor than the other prostaglandin receptors that carry out separate functions. The receptor specificity of selexipag theoretically reduces its side-effect profile [17,24].

#### 3.4.3. Evidence for Prostacyclins

Epoprostenol was the first FDA-approved therapy for IPAH (1995). High-dose epoprostenol has been shown to potentially reverse vascular remodeling in pulmonary arterioles by reduction of medial hypertrophy. Early randomized control trials (RCTs) provided evidence for improved exercise tolerance, 6MWD, and hemodynamics over 12 weeks. These studies were conducted in IPAH, PH with associated scleroderma, and WHO FC III–IV [17,22,23]. Epoprostenol remains one of the few therapies that reduce mortality in PAH [23]. The drug showed a relative risk reduction with a 70% decrease in mortality in PAH patients in a meta-analysis [15]. The need for continuous IV infusion limits the benefit regarding patient quality of life.

Treprostinil was first studied as a SQ infusion in a placebo-controlled RCT of 470 patients over 12 weeks [30] and was FDA-approved in 2002 for PAH FC II–IV. A later study found that SQ treprostinil improved hemodynamics, symptoms, and quality of life, and produced moderate but significant improvement in exercise capacity [31]. The 2012 study also found there was a survival benefit of 57% over a long period of 9 years. A placebo-controlled, randomized multicenter study with 235 PAH patients already on either oral bosentan or sildenafil (TRIUMPH-1, 2010) showed that the addition of inhaled treprostinil improved 6MWD [31]. Oral treprostinil has demonstrated improved exercise capacity as monotherapy in those not requiring high-risk therapy. The FREEDOM C1 and C2 trials failed to show exercise capacity improvement in PAH patients with oral treprostinil as add on therapy, but the follow-up FREEDOM-EV trial in 2021 did show in increased TTCW in patients already on ERA or PDE5i [17,32]. A dry powder inhaled (DPI) delivery system was investigated in the BREEZE trial and was recently approved by FDA in 2022 for PAH patients [33]. The BREEZE trial consisted of a 3 week open-label trial in PAH patients currently on inhaled treprostinil solution switched to the DPI form and was found to be safe and well tolerated.

Inhaled iloprost was FDA-approved for PAH monotherapy in 2004 following the results of the AIR trial, and randomized trials with it showed increased 6MWD, decreased PVR, and improved quality of life after 12 weeks versus placebo [17,22,23]. Since then, the evidence for inhaled iloprost has been mixed as two RCTs (COMBI and STEP) evaluated the effects of inhaled iloprost as add-on therapy in PAH patients already taking bosentan and showed conflicting data on outcomes [17].

Selexipag showed improved hemodynamics, with improvement in both PVR and cardiac index in a 17 week phase II study [17,23]. The phase III GRIPHON trial compared selexipag therapy to placebo in 1156 patients who were either treatment-naïve patients or were on an oral ERA and/or PDE5i therapy. The trial showed a relative risk reduction of in the primary composite endpoint of death or complication due to PAH [24]. Selexipag was FDA-approved in 2015 [17,34].

A novel oral selective prostacyclin receptor agonist, ralinepag, was shown to reduce PVR in PAH patients on mono or dual therapy in a phase II clinical trial. ADVANCE OUTCOMES is a phase III RCT currently investigating ralinepag’s effectiveness when added to approved therapies with a primary endpoint of time to clinical worsening (TTCW) [17,21,35].

#### 3.4.4. Endothelin Receptor Antagonists

Endothelin receptor antagonists (ERAs) either block ET-A receptors of both A and B. Currently, bosentan, ambrisentan, and macitentan are the three ERAs that are FDA-approved for the treatment of PAH.

**Figure 3:**
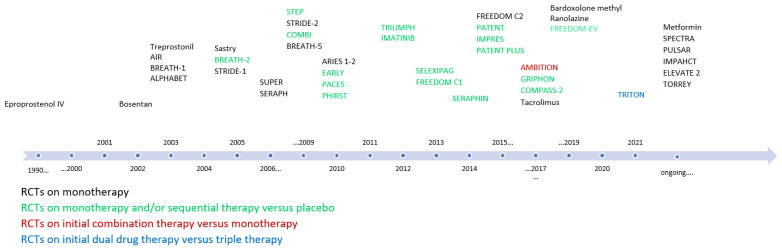
Timeline of RCTs published in PAH treatment.

Bosentan is a dual ERA (ET-A and ET-B) and represents the first oral PAH therapy that received FDA approval in 2001 (Figure 3) [20]. A major side-effect of bosentan is the reversible elevation of transaminases, requiring periodic assessments of liver enzymes and other laboratory tests while on therapy. This adverse effect has been reported in approximately 10% of patients on the medication and is believed to be the consequence of cellular bile acid accumulation [17,20]. Other side-effects of bosentan treatment include headache, nasal congestion, and edema. Bosentan also has numerous drug-drug interactions as it is a CYP 450 inducer. Ambrisentan is a selective ERA (ET-A) FDA-approved in 2007 that does not require hepatic monitoring but still retains the other side-effects specific to ERAs [28]. Ambrisetan’s side-effects include increased frequency of nasal congestion, peripheral edema, and headache, likely due to systemic vasodilation [36]. Ambrisentan is available in once daily oral doses of 5 mg and 10 mg. Macitentan is a dual ERA that was created on the basis of the structure of bosentan but designed for better safety and efficacy. This once-daily pill does not show the hepatotoxicity or drug-drug interactions of bosentan and appears to be more effective in improving exercise capacity [37]. The main side-effects seen in clinical trials were nasopharyngitis, headache, and anemia. Anemia with hemoglobin less than 8.0 g/dL was observed in 4.3% of patients on macitentan treatment in one clinical trial [37].

#### 3.4.5. Evidence for the ERAs

RCTs with bosentan have shown improvement in hemodynamic, 6MWD, echo parameters, and FC [38]. The BREATHE-1 RCT of 21 3FC III and IV PAH patients demonstrated an improvement in 6MWD and TTCW over 16 weeks. A series of BREATHE trials have since been conducted with both monotherapy and combination with IV epoprostenol (Figure 3) [38]. The EARLY RCT with 168 patients over 6 months showed increased 6MWD and improved PVR as monotherapy in FC-II PAH [17]. The more recent COMPASS-2 trial examined the benefit of adding a second oral agent in PAH patients already on monotherapy with sildenafil. The COMPASS-2 trial added bosentan to those on sildenafil monotherapy in a double-blind RCT vs. placebo. The composite primary endpoint was the time to the first morbidity/mortality event; bosentan addition was not found to be superior to monotherapy with sildenafil alone [38].

Ambrisentan is another once daily oral medication that was studied in the ARIES trials. ARIES-1 and 2 investigated Ambrisentan as monotherapy vs. placebo and showed improved hemodynamics and exercise capacity in PAH (IPAH and CTD). AIRES-E was a 2 year extension to these trials that showed sustained benefits with the medication [17,19,39]. The AMBITION trial investigated upfront combination oral therapy (ambrisentan and tadalafil) vs. initial monotherapy (ambrisentan or tadalafil) in low-risk treatment-naïve patients. The combination group showed a reduction in the risk of first clinical event worsening by 50% with a primary composite endpoint of TTCW [40]. Further post hoc analysis demonstrated similar effects in various subgroups. This was the first trial to provide clear evidence of a survival advantage with initial oral combination therapy over monotherapy in FC-II-III PAH and granted ambrisentan and tadalafil level 1A evidence for use as initial combination therapy [17,19]. This resulted in a paradigm shift wherein upfront combination therapy in PAH became the standard of care in sicker patients rather than the stepwise approach that was common prior to this, which has translated into improved morbidity and mortality.

Macitentan was originally FDA-approved as a once-daily oral medication for FC II-III following the SEPHARIN trial, a large phase III RCT (*n* = 742) with PAH patients, published in 2013 [17,20,34]. This was the first ERA study with a primary endpoint of morbidity and mortality. Macitentan was studied vs. placebo both as monotherapy and in combination with a PDE5 inhibitor, and a mortality benefit was demonstrated in both groups. Patients on prostacyclin therapy were excluded. Macitentan was the only therapy other than epoprostenol at the time shown to have a mortality benefit [17]. For this reason, cost considerations aside, macitentan may have a superior efficacy and safety profile when selecting from the ERA class. A phase III RCT (UNISUS) is currently underway comparing macitentan at 75 mg vs. current 10 mg dosing with a primary endpoint of TTCW [21].

A 2019 retrospective cohort study compared initial monotherapy with an ERA vs. PDE5i and showed no difference in the primary outcome of mortality between the two classes. However, age was an important effect modifier as ERA monotherapy was associated with decreased mortality in younger patients [21,35].

#### 3.4.6. The Nitric Oxide pathway

Patients with PAH typically have both decreased levels of nitric oxide synthase and overproduction of PDE5 in the pulmonary vasculature. Since NO’s potent vasodilator effects stem from cGMP production that relaxes SMCs, therapies in the NO pathway focus on increasing levels of cGMP. Most medications in this pathway directly interact with the enzyme superfamily of phosphodiesterases which normally inactivate and decrease cGMP levels. In PAH, overexpression of PDE5 in lung vasculature leads to endothelial dysfunction through over-degradation of cGMP. Phosphodiesterase inhibitors (PDE5is) stop the degradation of cGMP, leading to pulmonary arteriolar vasodilation [8,17]. Sildenafil and tadalafil are the two currently approved PDE5is for use in PAH. Both oral medications can be given up to three times daily. Both these medications carry class-specific side-effects including headache, flushing, nasal congestion, gastrointestinal disorders, and myalgias [17].

The soluble guanylate cyclase (sGC) stimulators were more recently developed specifically for PAH treatment. These oral medications directly increase cGMP levels by enhancing production through guanylate cyclase stimulation. Although they act independently from NO, they also increase guanylate cyclase sensitivity to NO, adding to their vasodilatory effects. Riociguat is the only approved medication in this class. sGC stimulators and PDE5is should not be used at the same time due to hypotensive effects [17,41].

#### 3.4.7. Evidence PDE5i and sGC simulators

Sildenafil was shown to improve 6MWD, symptoms, and hemodynamics in a 12 week RCT (SUPER-1) as monotherapy vs. placebo [17,26]. In an open-label extension (SUPER-2) of this trial, the long-term efficacy of sildenafil therapy was demonstrated. Sildenafil is dosed from 20 to 80 mg TID, but the most long-lasting efficacy is seen at the 80 mg dose. The PACES trial showed increased exercise capacity and TTCW with the addition of sildenafil in high-risk PAH patients who were on IV epoprostenol [17,19,42].

Tadalafil showed an increased 6MWD in the 16 week extended PHIRST trial that had 405 PAH patients (Figure 3). This study compared both treatment-naïve patients and patients taking bosentan. Patients were randomized to placebo or 2.5, 10, 20, or 40 mg once daily of tadalafil. Maximal efficacy was seen at 40 mg. As mentioned, the AMBITION trial used a combination of tadalafil and ambrisentan to improve TTCW in FC II-III. [17,19,42,43].

Riociguat’s safety and efficacy were established in the PATENT-1 RCT with 400 PAH patients. Riociguat was dosed at 2.5 mg three times daily and demonstrated improved hemodynamics and TTCW. An open label extension study PATENT-2 showed the improved effects of the original studies continued for over 2 years [21]. The RESPITE study looked at patients who did not have an adequate response on PDE5i by replacing the PDE5i with Riociguat. This 24 week, open-label uncontrolled study showed an increased 6MWD and WHO FC improvement in 54% of the participants. The REPLACE RCT trial evaluated the strategy of switching from a PDE5i to riociguat in PAH patients who were at intermediate risk of 1 year mortality. In the 226 patients enrolled, clinical worsening occurred in 1% of patients in the riociguat arm vs. 9% of patients in the PDE5i arm (odds ratio 0.10 [0.01–0.73]; *p* = 0.0047) [17,41,44]. Thus, switching between different classes of NO pathway drugs may be an option when one class does not yield the expected clinical benefit; riociguat may provide an option for treatment escalation in such patients.

#### 3.4.8. Monotherapy vs. Combination Therapy

For PAH patients stratified as low-risk, treatment-naïve patients in FC I–II, guidelines historically recommended monotherapy with a single oral agent, with the sequential addition of medications if clinical improvement was not met. A meta-analysis of 35 RCTs demonstrated a benefit of PAH specific monotherapy over placebo with respect to 6MWD and quality of life. There are currently no studies comparing different classes of medications as monotherapy available [28,29].

In more recent years, there has been a paradigm shift toward investigating combination versus monotherapy in RCTs, favoring more upfront aggressive strategies. The AMBITION trial led to a change in the ESC/ERS and CHEST guidelines to adopt the combination of ambrisentan and tadalafil as the preferred regimen in FC II-III PAH [40]. Sequential therapy was not included in this study and, thus, was not directly compared. Notably, the 2015 COMPASS-2 trial investigating bosentan and sildenafil vs. sildenafil monotherapy did not demonstrate a benefit of dual therapy vs. monotherapy but was not adequately powered and had a high attrition rate [17,19,45].

In 2021, the first study comparing dual therapy to triple therapy (macitentan, tadalafil, and placebo vs. macitentan, tadalafil, and selexipag) in treatment-naïve PAH patients was published (Figure 3) [46]. TRITON was a 26 week RCT in PAH FC II–III patients with a primary endpoint of change in PVR. The study did not find a significant difference between the two treatment arms and did not support triple oral therapy over dual oral therapy in this population. However, there was a signal of benefit in the triple therapy arm on longer follow up and this remains to be conclusively established in future trials. In high-risk patients or those refractory to treatment, initial triple therapy with the inclusion of IV prostacyclin therapy is recommended [19,21].

### 3.5. Novel Treatment Therapies

#### 3.5.1. BMPR2 Modulators

The BMPR2 gene has been identified as a major role player in the pathophysiology of PAH. Loss-of-function mutations in this gene lead to unopposed TGF-β signaling. Multiple therapies are under investigation to modulate the dysfunction involved in this family of receptors. Sotatercept has been designed to stabilize the imbalance of BMPR2 and TGF-β signaling in this pathway [19]. The first of its class, it is a fusion protein that works as a selective ligand trap for TGF-β molecules. This leads to TGF-β suppression and enhances BMPR2 signaling. A phase II double-blind placebo-controlled study (PULSAR) was performed in FC II–III PAH patients [47]. In this 24 week trial, SQ sotatercept was administered every 21 days on top of standard PAH treatments vs. placebo and showed reduced PVR (primary endpoint) and increased 6MWD. These effects were shown in patients taking monotherapy or dual oral therapy and triple therapy with prostacyclin infusion therapy. Further phase III trials are being conducted (STELLAR) in FC-III PAH patients [21]. In the PULSAR trial, sotatercept showed a reduction in PVR at 24 weeks, as well as reduced NT-pro BNP levels [48].

FK506 (known as tacrolimus) is an FDA-approved calcineurin inhibitor used for both the induction and the maintenance of postoperative immunosuppression. Research has shown that tacrolimus also acts as a potent BMPR2 activator that reverses endothelial dysfunction in experimental PH in mouse models. Another mouse model demonstrated reduced RV fibrosis and occlusive vasculopathy via the BMP pathway; this effect was independent of its immunosuppressive effect [17,19,21]. Such an effect might help preserve RV–PA coupling and improve RV function over time. A small compassionate trial in three end-stage PAH patients showed improvement in 6MWD and FC in a 12 month period. A phase IIa RCT (TransformPAH) with 23 PAH patients investigated tacrolimus targeted to achieve three separate serum levels over 16 weeks [17,19,21,49]. This trial showed that tacrolimus was safe and well tolerated in patients on current guideline directed PAH therapy, but it was not powered to examine efficacy. More genetic targets have been identified but are still early in the research process.

#### 3.5.2. Platelet-Derived Growth Factor (TK Inhibitor Imatinib)

**Figure 4:**
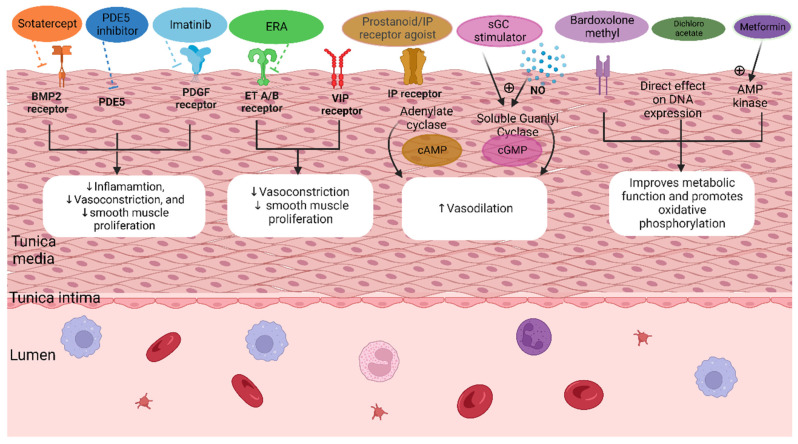
Mechanism of action (MOA) of specific PAH therapies at the receptor level. ↓ = decreased, ↑ = increased.

The platelet-derived growth factor signaling pathway (PDGF) is overexpressed in the pulmonary vasculature of patients with PAH, leading to endothelial dysregulation and SMC proliferation (Figure 4) [19,21]. Tyrosine kinases have been implicated in this pathway to contribute to the vascular remodeling in PAH [17,19]. Studies have also shown crosstalk between the PDGF signaling pathway and the BMPR2 axis [50] using transcriptomic and proteomic analysis (Figure 4).

Tyrosine kinase inhibitors such as imatinib are being investigated as a pharmacologic option in PAH. The IMPRES trial studied oral imatinib in high-risk PAH patients on dual or triple therapy in a phase III RCT of 24 weeks (*n* = 150) and showed improvement in 6MWD and PVR [51]. High rates of serious adverse events, including subdural hematomas in patients on anticoagulation have halted further studies with imatinib in the oral form. Inhaled imatinib is now enrolling a phase II/III trial called the IMPAHCT trial. This trial will evaluate the safety and efficacy of AV-101 (dry powder inhaled imatinib) versus placebo with a primary endpoint of change in PVR at 24 weeks. The primary endpoint of the phase III portion of the trial will be the change in 6 min walk distance (6MWD) over 24 weeks. Seralutinib is a selective kinase inhibitor that targets the PDGF, among other signaling cascades, and is specifically designed for the treatment of PAH. Animal trials have shown the reversal of vascular remodeling associated with PAH with the administration of seralutinib [52]. Seralutinib is delivered as a dry powder inhaler; it passed phase 1 clinical trials and, in 2021, began enrollment in a phase II trial where the primary endpoint is a change in PVR on RHC at 24 weeks [52,53] Other tyrosine kinase inhibitors are under investigation, but none are yet in phase II trials.

#### 3.5.3. Mitochondrial Dysfunction and Oxidative Stress

Another class of medications for PAH is based on the observation that pulmonary vascular cells in patients with PAH switch their metabolism from mitochondrial oxidative phosphorylation to glycolysis known as “the Warburg effect”. This metabolic dysfunction leads to abnormal cell proliferation and resistance to apoptosis [19,54]. Reactive oxygen species (ROS) levels increase due to changes in aerobic respiration; this results in increased vascular tone. On the other hand, nuclear factor erythroid 2-related factor 2 (Nrf2) activation promotes mitochondrial respiration, reduces ROS, decreases inflammation, and improves metabolic function. Bardoxolone methyl is an oral Nrf2 transcription factor activator with promising evidence. In the phase II LARIAT study, bardoxolone added to background therapy showed improvement in 6MWD at 16 weeks. However, phase III studies (RANGER and CATALYST) were halted due to COVID-19 [19,21].

Dichloroacetate (DCA) has two proposed mechanisms of action that may be beneficial in PAH. First, it inhibits pyruvate dehydrogenase kinase, leading to the increased activity of pyruvate dehydrogenase which restores preferential oxidative phosphorylation. This, in turn, reduces the oxidative stress that leads to ROS formation and vascular remodeling in the pulmonary arterioles. The second proposed mechanism involves the family of potassium gated ion channels (kv) expressed in pulmonary artery SMCs [17,19,21]. This kv family of receptors shows decreased expression during hypoxic conditions, which can lead to increased vasoconstriction. These kv receptors are expressed in low levels in some forms of PAH which may contribute to vascular remodeling via SMC proliferation and reduced apoptosis. DCA can increase the expression of kv receptors in isolated pulmonary artery SMCs in vitro. A 16 week phase I trial with DCA in PAH patients (n = 20) demonstrated safety and showed improvement in PA pressures and PVR [17,19].

Another medication that could potentially be repurposed for PAH is metformin. Insulin resistance has been shown to correlate with morbidity and mortality in PAH. However, it is still uncertain whether there is a causal relationship [13,15]. Mouse models have also demonstrated favorable effects of metformin on the prostacyclin, ET, and NO signaling pathways [55]. AMP kinase activation by metformin inhibits SMC proliferation caused by a dysfunctional ET pathway and can also stimulate nitric oxide synthase. Hemodynamic effects in mouse models have not been consistently positive; however, phase II human trials are currently underway [55].

#### 3.5.4. Renin–Angiotensin–Aldosterone System

Aldosterone is a steroid hormone involved in the renin–angiotensin–aldosterone system responsible for regulating systemic blood pressure. Aldosterone binds to mineralocorticoid receptors, including in the heart, kidney, and pulmonary vasculature. Aldosterone alters gene transcription to increase water retention, cardiac fibrosis, and activation of the sympathetic nervous system. Antagonists of this receptor, such as spironolactone, are used to reduce cardiac fibrosis and adverse remodeling in heart failure with reduced ejection fraction. This agent may have potential utility in some forms of PH [8,19,21]. Serum levels of aldosterone are elevated in PAH patients. Other studies have shown a positive correlation between aldosterone levels and PVR and an inverse relationship between aldosterone levels and CO [8,19]. The question of whether there are structural benefits to mineralocorticoid receptor antagonists (MRAs) in PAH aside from the fluid balance effect is currently being explored. An open-label RCT is currently underway investigating spironolactone in PAH patients, with 6MWD and TTCW as endpoints [56].

#### 3.5.5. Other Novel Pharmacological Targets

As more molecular pathways in the pathogenesis of PAH are discovered, new targets for drug therapy have also been identified, although many of these therapies are still early in the clinical trial process. Rho-kinases are intracellular modulators highly expressed in arteries. The Rho-kinase pathway has been identified as a key player in the vascular remodeling in PAH. Rho-kinase inhibitors have been developed to mediate the effects of this remodeling and help increase the expression of nitric oxide synthase [17,19,21]. These are currently entering phase II trials.

Vasoactive intestinal peptide (VIP) is a neuroendocrine hormone that has been shown to cause SMC relaxation, neutralize vasoconstrictors, and inhibit SMC proliferation. Low serum concentrations of VIP have been seen in the serum of PAH patients [17,19,21]. Extended-release VIP analogs have been developed as a potential treatment. Pemziviptadil is given as an SQ injection and is currently being explored in a phase II trial for PAH. Rodastristat ethyl is a peripheral inhibitor of tryptophan hydroxylase 1, the rate-limiting enzyme in the production of serotonin in peripheral circulation. Locally produced serotonin from lung tissue and arterial endothelial cells induces excessive growth of pulmonary artery smooth muscle cells. The unchecked growth of these cells drives remodeling of pulmonary arteries that reduces the diameter and flexibility of the arterial lumen in PAH [57,58]. ELEVATE 2 is a phase IIb clinical trial currently enrolling with a primary endpoint of change in PVR at 24 weeks [57,58].

## 4. Group 2 PH: Pulmonary Hypertension Due to Left-Heart Disease (PH-LHD)

Left-heart disease (LHD) is the most common etiology of pulmonary hypertension; it is estimated that approximately 70% of PH is due to LHD. It can be associated with any form of LHD including heart failure with reduced ejection fraction (HFrEF), heart failure with preserved ejection fraction (HFpEF), left-sided valvular disorders, and inflow/outflow tract abnormalities.

### 4.1. Definition

PH-LHD is defined as an mPAP ≥ 20 mmHg with a PCWP ≥ 15 mmHg. Elevated PCWP is the defining feature of this subgroup and suggests that the driver of this disease is the transmission of elevated left-atrial pressure (LAP) to the pulmonary venous vasculature. PH-LHD can be further classified into two subtypes, isolated postcapillary PH (Ipc-PH) and combined post- and precapillary PH (Cpc-PH) [59,60]. Both groups involve the passive transmission of pressure from the left atrium, with the latter implying intrinsic pulmonary valvular disease that develops in longstanding cases.

### 4.2. Epidemiology

The true prevalence of group 2 PH is poorly understood, due in part to the various diagnostic modalities used. Most epidemiological studies have utilized echocardiographic-derived PA systolic pressure rather than catheter based measured mPAP, and echo-based diagnosis has been criticized for overestimating the prevalence of PH. Regardless of the modality used, the estimated prevalence of PH is 60–70% in HFrEF patients and approximately 50% in the HFpEF population [59,60].

### 4.3. Pathophysiology

The primary event leading to PH-LHD is the passive transmission of elevated LAP into the pulmonary circulation driven mainly by LV dysfunction. This passive process leads to Ipc-PH. With time, persistently elevated LA pressure leads to pathologic structural and biochemical changes in the pulmonary vasculature, eventually leading to Cpc-PH. The pathophysiology of PH-LHD is complex and involves multiple interrelated mechanisms briefly summarized below.

Initially, increased LAP from LHD results in a passive transmission of pressure to the pulmonary vasculature, resulting in Ipc-PH. Secondly, persistent elevation in the pulmonary venous system leads to pathologic remodeling of the pulmonary arterial system, characterized by medial hypertrophy and intimal fibrosis. Of note, the typical plexiform lesions of PAH are absent. Thirdly, changes to the pulmonary vasculature exacerbate endothelial dysfunction, with an ensuing decrease in NO production and an increase in ET production, ultimately leading to impaired vasodilation and an increase in PVR. Lastly, long-standing elevation in the PAP and RV afterload can result in maladaptive changes in RV structure, leading to RV dysfunction and eventually RV failure [5,43,44,45]. Patients with PH-LHD often have RV functional abnormalities despite normal LV systolic function that occur not only from elevated left-heart pressure and passive transmission to the pulmonary venous circuit but also likely involve some element of the development of pulmonary arteriolar disease over time [3]. PH in patients with LHD may also be due to other coexisting causes, including undetected concomitant CTEPH or PAH. RV function is an important predictor of mortality in these patients, as it is in other forms of PH [7,59,60,61].

### 4.4. Treatment

Therapy targeting the underlying LHD remains the most established strategy for PH-LHD. In the case of heart failure, guideline directed medical therapy should be titrated to target doses [62]. Device therapies such as cardiac resynchronization therapy and continuous PAP monitoring devices remain reasonable strategies in refractory cases or progressive disease. Surgical or transcatheter intervention for valvular disease and ischemic heart disease should be pursued when indicated. Optimization of comorbidities including obstructive sleep apnea (OSA) and chronic obstructive pulmonary disease (COPD) should also be addressed [63]

Given the prevalence of PH-LHD and its associated morbidity and mortality, repurposing PAH drug therapies to target this group has been investigated, but there has been no clear benefit, and some cases have been shown to have increased mortality and significant adverse effects. For example, recent data have shown that there is no role for nitrates or sildenafil in Ipc-PH as detailed below.

#### 4.4.1. Epoprostenol

The FIRST Trial studied the use of epoprostenol in 471 patients with HFrEF. Although there were improved hemodynamic markers such as cardiac index (CI) and PCWP, the trial was terminated early due to increased mortality in the treatment group [64]. Currently, there is no strong evidence to support the use of epoprostenol in PH-LHD.

#### 4.4.2. Endothelin Receptor Antagonists (ERA)

Multiple trials have studied the use of ERAs in HF, with the most studied medication being bosentan. The Enable trial studied 1613 HFrEF patients; the use of bosentan did not improve mortality or hospitalizations and was associated with worsening fluid retention [34,65]. The EARTH trial studied darusentan in HFrEF patients, and failed to show improved outcomes [65]. The MELODY-1 trial evaluated macitentan in PH-LHD; the treatment group experienced more adverse events, with no improvement in endpoints [66]. Currently, major studies have shown no benefit to the use of ERA in heart failure patients.

#### 4.4.3. Phosphodiesterase 5 Inhibition

There have been several studies investigating the use of PDE-5 inhibitors, with no unifying recommendations or compelling evidence to support their use. In the RELAX trial, sildenafil did not improve the 6 min walk test or quality of life in 216 HFpEF patients. In a meta-analysis examining nine RCTs involving 612 patients with HF, there were improved hemodynamic parameters in HFrEF patients, with no difference in quality-of-life measurements. In a more recent study involving 40 patients with Cpc-PH, there was an improvement in subjective dyspnea, 6 min walk test, and hospitalizations [67]. Given the number of studies with mixed results, stronger evidence is required prior to recommending for or against the routine use of PDE5i in PH-LHD.

#### 4.4.4. Soluble Guanylate Cyclase Stimulators

Soluble guanylate cyclase stimulators increase the production of NO, which has vasodilatory and antifibrotic effects. Recent studies have investigated riociguat in patients with PH with left-heart failure. RCTs by Bonderman et al. demonstrated no significant change in the primary endpoint of decreased mPAP but did show an improvement in hemodynamic markers such as CI, PVR, and stroke volume [68]. Other studies have investigated the use of soluble guanylate cyclase stimulators (vericiguat) in heart failure patients, e.g., the SOCRATES-PRESERVED (LVEF ≥ 45%) and SOCRATES-REDUCED (LVEF < 45%) trials [69]. Neither study specifically investigated their use in PH patients, and neither study met the primary endpoint of a reduction in NT-pro BNP levels [70].

#### 4.4.5. Metformin

Metformin is one the most widely used medications for the management of type 2 diabetes mellitus. T2DM and HF are closely linked disease processes, and the prevalence of T2DM among HF patients is approximately 30%. Several studies have evaluated the benefit of metformin in diabetics with improved microvascular outcomes with good glycemic control [55,71]. Preclinical models have provided mechanistic evidence supporting metformin’s use in the PH population with left-heart failure. Given the lack of available therapies for PH-LHD, there are ongoing trials studying the effect of metformin on PAH and PH in HFpEF [71].

## 5. Group 3 PH: Respiratory Diseases and Treatments

PH can develop because of common parenchymal lung diseases such as COPD, interstitial pulmonary fibrosis, or OSA. More rarely, PH can be seen in cystic fibrosis and high-altitude exposure. This group is the second most common form of PH; while there is increased mortality in this population, it is unclear whether PH contributes directly to the increased mortality or whether it is an independent marker for the severity of lung disease progression. Group 3 patients tend to have a higher mortality than Group 1 patients [72]. PH should be sought for and excluded in lung disease when signs and symptoms of right-heart failure develop.

### 5.1. Pathophysiology and Etiology

The pathogenesis of PH in lung disease is multifactorial with several inciting mechanisms. The most common mechanism begins with hypoxia due to lung disease as many chronic lung diseases cause a reduced diffusing capacity of the lung for carbon monoxide. Hypoxia causes contraction of the pulmonary vessels and leads to vascular remodeling via ET and serotonin. If the hypoxia becomes chronic, it is only partially reversible with supplemental oxygen. There is a longstanding belief that the changes in lung anatomy and physiology lead to the vascular changes seen in group 3 PH via direct fibrosis or loss of surface area of the arterial bed, causing increased PA pressure [73]. Recent evidence suggests that this process is more dynamic; vascular remodeling has been found in tissue samples in patients with lung disease even in areas of the lung that are disease-free, implying hormonal or cytokine mediated changes. The severity of lung disease also does not seem to correlate with PH severity [74,75]. Thus, the pathogenesis of group 3 PH seems to be more complex and likely also involves inflammatory cascades and growth factors, as is the case in group 1 PH.

### 5.2. Classifications

Almost 90% of COPD patients have a mPAP > 20 mmHg but only 5% will develop a mPAP > 40 mmHg, making severe PH rare in COPD. A unique feature of Group 3 PH is out-of-proportion RV dysfunction compared to the pulmonary vascular disease, especially when compared to other PH groups. Some possible explanations are that COPD patients tend to have already existing RV dysfunction [74]. Another reason is that group 3 has a higher proportion of males than group 1, and males have a lower average RV ejection fraction according to some studies.

Similarly, OSA also commonly causes only mild PH and rarely progresses to severe PH. Fortunately, the mild form is reversible with treatment such as noninvasive nocturnal positive pressure ventilation (such as CPAP—continuous positive airway pressure devices) [75].

### 5.3. Diagnosis

Since the presenting symptoms of chronic lung disease and PH overlap (dyspnea, hypoxia, and fatigue), a high index of suspicion is required to make the diagnosis of group 3 PH. Signs that should immediately prompt a PH workup in these patients are signs of RV failure and enlarged PA on imaging. The workup for PH should ideally be conducted while patients are stable and not in a current exacerbation. Group 3 PH diagnosis still follows the same diagnostic pathway as other groups, ultimately requiring an RHC. Group 3 is a precapillary form of PH; thus, PCWP will be less than 15 mmHg on RHC [72,73]. For those who have only mild symptoms or mildly increased PA pressures on echo, such as those with pre-existing OSA, RHC may not be necessary as these symptoms are reversible with noninvasive ventilation. Pulmonary function tests are a requirement in the diagnosis of parenchymal lung disease; thus, these are always indicated. All other causes of PH also need to be ruled out with appropriate testing [13].

### 5.4. Treatment

The aim is to improve lung function and correct hypoxemia. This means treating the underlying cause of the lung dysfunction, which is disease-specific. The role of vasodilators is more complex with mixed data [72]. In COPD with PH, bosentan and sildenafil have been studied and shown to improve hemodynamics. Unfortunately, these hemodynamic improvements have failed to show increased exercise capacity or other meaningful benefits [76]. For example, in the BUILD-3 event-driven randomized controlled placebo trial of bosentan in idiopathic pulmonary fibrosis (IPF), the primary endpoint of worsening of IPF or death was not met [77,78]. Similarly, the B-PHIT trial showed no difference in symptoms, functional capacity, or hemodynamics at 16 weeks between bosentan and placebo in patients with PH and fibrotic idiopathic interstitial pneumonia [79].

The MUSIC phase II trial, completed in 2013, with macitentan versus placebo in the treatment of PH caused by idiopathic pulmonary fibrosis, did not meet its primary endpoint of a positive change in forced vital capacity [80]. Ambrisentan was also investigated in idiopathic pulmonary fibrosis in a similarly designed trial that was prematurely discontinued as the interim analysis showed a low likelihood of efficacy [81]. Sildenafil also showed no benefit in IPF in the STEP-IPF trial [82]. Riociguat has also been studied in PH due to idiopathic interstitial pneumonia where bosentan had failed to show a benefit. In a phase IIb study [83] in 147 patients (RISE-IIP trial), riociguat was also not beneficial, showing worsening of interstitial lung disease and no improvement in 6MWD. However, the INCREASE trial in ILD patients with coexisting PH examined inhaled treprostinil vs. placebo and found an increased exercise capacity over a 16 week period [76,84]. On the basis of these data, treprostinil was recently approved in ILD PH.

## 6. Group 4 PH: CTEPH

### 6.1. Pathogenesis and Etiology

CTEPH is a complication that may rarely occur after PE and is characterized by PH that persists for more than 3 months following effective anticoagulation therapy. Even though it is believed to reflect PH occurring from unresolved thromboemboli in the pulmonary arterial tree, more than one-third of cases of the index venous thromboembolism episodes in these patients are clinically silent with patients reporting no prior history of clinically evident pulmonary emboli [85]. Other factors such as in situ thrombosis or inflammatory responses to such thrombi also likely play a role in its pathogenesis [85,86]. It is, thus, characterized by thromboembolic material in the distal Pas, which leads to altered vascular remodeling. Prospective studies where right-heart catheterization was performed on patients with prior symptomatic pulmonary emboli showed that only 0.4% to 6.2% of patients developed CTEPH. Several inflammatory and thrombophilia markers have been found in CTEPH patients. Variable gene expression has also been demonstrated in pulmonary artery endothelial cells from patients with CTEPH [86]. Why some people with thromboembolic events develop CTEPH and others do not is unclear.

### 6.2. Making the Diagnosis

Testing for CTEPH starts with initial assessment with an echocardiogram. If pulmonary hypertension is suspected in a patient with prior PE, a ventilation/perfusion scan is performed to assess for chronic thromboemboli. A normal V/Q scan can exclude CTEPH with a sensitivity of 90–100% and specificity of 94–100% [86]. About 30–50% of patients with documented CTEPH will not have a clear history of prior venous thromboembolism. Thus, a VQ scan is a standard test indicated in most patients with PH undergoing workup with a standardized diagnostic algorithm.

### 6.3. Surgical Options and Indications

Pulmonary thromboendarterectomy (PTE) is the only definitive and potentially curative therapy for CTEPH. Between 15% and 51% of patients have residual PH following the procedure [86]. PEA is a major thoracic surgery performed under extracorporeal circulatory hypothermic arrest, and patients undergo thorough evaluation prior to being deemed appropriate candidates. For PTE to be performed, the thrombus must be accessible by surgery, and patients are screened for any comorbidity that would prohibit the surgery (lung function test and coronary artery disease screening). Some factors that have been shown to have less favorable outcomes include FC IV, significant concomitant lung or heart disease, right-heart failure, PVR > 1200 dynes·s/cm^−5^ (15 wood units), and absence of appreciable lower lobe disease. Perioperative mortality is between 2% and 4% [86]. Following PTE, there is often a dramatic improvement in PAPs and PVR. In one study, the PVR was reduced immediately postoperatively by nearly 70% [87]. While there might not always be complete resolution of PH, the severity decreases, and patients report improvement in symptoms. In one study, the 3 year survival of patients who had PTE was reported at 90% vs. 70% in patients who did not have surgery [86,87].

### 6.4. Medical Management and Current Therapies in Nonsurgical Candidates

Medical therapy is noncurative and is reserved for those patients who do not qualify for PTE. The first step in the management of CTEPH is initiation of anticoagulation therapy. The anticoagulation therapy is based on provider preference. Studies reviewing the superiority of the newer direct oral anticoagulants (DOACs) versus vitamin Ka antagonists (such as warfarin) are limited.

In patients with FC II or III symptoms, initiation of riociguat is recommended. Studies have demonstrated improvement in exercise capacity, FC, and pulmonary hemodynamics with CTEPH patients on riociguat (CHEST-1 Trial). A follow-up study (CHEST-2) reported that prolonged therapy for up to 2 years with riociguat demonstrated persistent benefits. The BENEFIT trial was a placebo-controlled trial that enrolled 157 patients with either inoperable CTEPH or persistent CTEPH after PTE and evaluated the effectiveness of bosentan. The bosentan arm showed improvement in PVR and CI, but there was no improvement in exercise capacity [88].

The AMBER 1 trial looked at ambrisentan compared to placebo in patients with inoperable CTEPH. Although the study was terminated early due to lack of enrollment, there were positive trends in 6MWD, PVR, and NT pro-BNP reported with ambrisentan [89]. Macitentan was investigated in inoperable CTEPH in a phase II RCT (MERIT-1); results showed a significant improvement in PVR at 16 weeks [90]. In 2018, the CTREPH phase III RCT showed improved exercise capacity in inoperable CTEPH patients in FC III-IV treated with subcutaneous treprostinil [90,91]. A randomized trial of 19 patients with inoperable CTEPH showed that 12 weeks of sildenafil improved WHO FC and PVR. There was no difference in exercise capacity noted. Following the initial study, the control arm was then provided open-label sildenafil. At 12 months, patients had an increase in exercise capacity, symptom score, and PVR [42].

## 7. Group 5 PH: Unclear or Multifactorial Causes

Group 5 pulmonary hypertension consists of a group of diseases that lead to PH via a multitude of mechanisms. For many of these diseases, the incidence and treatment of PH are uncertain. The increase in vascular resistance can be secondary to vasoconstriction, inflammation, proliferative arteriopathy, shunting, chronic anemia, veno-occlusive disease, left-ventricular dysfunction, or valvular heart disease. When looking at the etiology of PH in these systemic diseases, one should look at each of these contributing factors individually.

### 7.1. Hematologic Disorders

In chronic myeloproliferative diseases such as polycythemia vera, essential thrombocythemia, and primary myelofibrosis, the etiology is believed to be due to chronic thromboembolic events (CTEPH), vascular remodeling, pulmonary veno-occlusive disease, tumor micro embolism, and drug-induced PH. The incidence of PH in this group is unclear but estimated to be between 36% and 48% according to case reports and small series.

Splenectomized patients have an increased risk of developing PH. After splenectomy, thrombotic and thromboembolic complications can occur. There have been reports of both CTEPH and IPAH in patients following spleen removal. The etiology is not well understood. One proposed mechanism is that the loss of the spleen’s filtering function allows abnormal red cells to remain in the peripheral circulation. This may lead to facilitation of the coagulation process.

PH associated with chronic hemolytic anemia secondary to hemoglobinopathies, such as sickle cell disease and thalassemia, also falls into Group 5. The incidence is reported to be around 10% in sickle cell patients. It is believed that endothelial injury, chronic inflammation, hypercoagulability, intravascular hemolysis, and altered bioavailability of NO contribute to the development of PH. There may also be some increase in the PCWP from left-heart dysfunction that can contribute.

### 7.2. Systemic and Metabolic Disorders

This grouping consists of diseases such as sarcoidosis-associated PH (SAPH) and pulmonary Langerhans cell histiocytosis PH (PLCH). There is an increased incidence of PH in patients with sarcoidosis, estimated to be between 5% and 28%, associated with increased morbidity and mortality. SAPH etiology is multifactorial, and, while the incidence is higher in those patients with fibrosis, it can also be independent of airflow restriction. SAPH may arise due to fibrotic lung involvement leading to pulmonary vascular bed injury, extrinsic compression of pulmonary vessels, vasculopathy/vasculitis of the pulmonary vasculature, pulmonary veno-occlusion, porto-PH, and left-ventricular dysfunction. Similarly, PLCH-PH is believed to develop from pulmonary vascular pathology, but the pathogenesis is not well understood.

### 7.3. Metabolic Disorders

This group consists of disorders such as thyroid disease, glycogen storage diseases, and Gaucher disease. The prevalence of PH in thyroid disease has been estimated to be around 20%. LV dysfunction can be seen in patients with thyroid disease and is likely one of the causes of development of PH. Enhanced catecholamine sensitivity, increased metabolism of intrinsic pulmonary vasodilators, and decreased metabolism of vasoconstrictors leading to increased pulmonary vasculature are other proposed mechanisms.

Glycogen storage diseases are a set of diseases that lead to defective glycogen synthesis or breakdown due to an enzyme deficiency. The etiology of PH development is unknown but is believed to stem from abnormalities in serotonin metabolism.

Gaucher disease (GD) is the most common lysosomal storage disease. It is due to a deficiency in the enzyme glucocerebrosidase that leads to buildup of glucocerebroside. Up to 30% of patients with untreated type 1 GD have been found to show signs of PH based on echocardiography. The mechanism is believed to be from pulmonary capillary infiltration with glucocerebroside, as well as bone marrow microemboli leading to vasculopathy. Splenectomy in these patients also leads to an increased risk of PH development.

### 7.4. Other Disorders

These disorders include chronic renal failure, fibrosing mediastinitis, tumor emboli, and mechanical vascular obstruction. The incidence of PH in these diseases has not been systematically evaluated. In chronic renal failure, the increase in pulmonary venous pressure from LV dysfunction, hypervolemia, and high output failure from anemia, AV fistula shunting, and vascular calcifications are all believed to play a role in development of PH.

### 7.5. Current Therapies

Myeloproliferative disorders: Cytoreductive agents (hydroxyurea/antiplatelets) are used to decrease risk of thrombosis and vascular events. No data support the role of vasodilators.

Splenectomy: Patients with evidence of CTEPH should be evaluated for possible PTE. If PTE is not possible, then treatment consists of anticoagulation and consideration of PAH-specific agents or lung transplantation. Two studies, one with bosentan and the other with riociguat, on patients with inoperable or persistent CTEPH after thromboendarterectomy showed an improvement in exercise capacity and in hemodynamics. There are no studies showing whether prophylactic anticoagulation can help prevent the development of CTEPH in this population.

Scleroderma (SCD): The current treatment of PH due to SCD is to treat the underlying disease. Prior studies looking at PAH therapies were not successful. The Walk-PHaSST study was a placebo-controlled trial looking at sildenafil in patients with SCD that was stopped early because of a higher incidence of hospitalization for vaso-occlusive pain crises with no suggestion of improvement in the sildenafil arm. The ASSET-1 and ASSET-2 studies aimed to assess the efficacy and safety of bosentan therapy in patients with SCD and PH. Both studies were terminated due to lack of efficacy, slow site activation, and withdrawal of sponsor support. Prostacyclin infusion was shown to acutely improve hemodynamics in a small subset of patients, but there are no studies showing efficacy of long-term prostacyclin therapy. A retrospective study looking at treatment with riociguat in SCD showed improvement in 6MWD, RVSP by echo, and FC. Vasodilator therapy is not routinely recommended for SCD-PH. It is usually initiated only in symptomatic patients with RHC confirmed FC II–IV PH without any signs of LHD. ERAs and prostanoids are recommended over other drugs in this group of patients [27,41].

Thalassemia: One trial performed on 10 patients with β-thalassemia and echocardiographically defined PH looked at efficacy of sildenafil in this population. After 12 weeks, there were no safety concerns with sildenafil and the tricuspid regurgitant jet velocity was decreased. There was no significant improvement in FC or the 6MWD test. There was a case report of patient showing improvement in the 6MWD and mPAP following bosentan for 1 year, but no hemodynamic studies were conducted to confirm its efficacy in a systematic manner [88,92,93].

SAPH: A double-blind placebo-controlled randomized trial with bosentan in patients with SAPH showed significant improvement in pulmonary hemodynamics [94].

PLCH-PH—Up to one-third of patients have a positive vasodilator response. Some uncontrolled small studies suggested improvement in mortality with PAH therapy, but there are no randomized studies supporting this approach [94].

Thyroid disease: In the setting of hyperthyroidism, treatment with antithyroid medications, radioactive iodine, or surgery has been shown to decrease the mPAP. Treatment with vasodilators has not been studied, and these drugs are not routinely recommended.

GSD-PH: Treatment consists of treatment of underlying disease with enzyme replacement. There are no large studies supporting the use of vasodilators.

GD-PH: PH in this population usually improves with enzyme replacement therapy. Severe cases have occasionally been treated with vasodilators but there are no larger, controlled studies supporting their use.

## 8. Conclusions

While new research increasingly sheds light on the causes and pathogenesis of PH and PAH, there are still large strides to be made in treatment of this disease. Newly discovered molecular pathways will hopefully become new therapeutic targets for novel drug therapies. Existing drug trials used endpoints that mainly assess symptom control; these trials typically showed an increase in the time to a decline in the quality of life. However, using symptom control as a primary goal of therapy does not evaluate whether these drugs ameliorate the root cause of the disease at the pathological or molecular level. Nonetheless, newer drugs are now in development that do indeed target the underlying pathogenetic mechanisms of the disease. Data-driven risk calculators and objective functional class schema have recently been adopted as the primary endpoints in clinical trials. This represents an encouraging shift because improvement in these endpoints has been shown to represent improvements in mortality [5]. Continued emphasis on reversing the multiple vascular, cellular, molecular, and metabolic abnormalities that are the hallmark of PAH will ultimately be rewarded with meaningful progress in treating this deadly disease.

## Data Availability

Data sharing not applicable.

## Abbreviations

6MWD	6 min walk distance
AV	Arterio-venous
BMI	Body Mass Index
BMPR	Bone morphogenic protein receptor
cAMP	Cyclic adenosine monophosphate
CCB	Calcium channel blockers
cGMP	Cyclic guanosine monophosphate
CI	Cardiac index
CO	Cardiac output
COPD	Chronic obstructive pulmonary disease
COX	Cyclo-oxygenase
Cpc	Combined post and precapillary
CTD	Connective tissue diseases
CTEPH	Chronic thromboembolic pulmonary hypertension
DCA	Dichloroacetate
DOACS	Direct oral anticoagulants
ERA	Endothelin receptor antagonist
ET	Endothelin
FC	Functional class
FDA	Food and Drug Association
GD	Gaucher disease
HFpEF	Heart failure with preserved ejection fracture
HFrEF	Heart failure with reduced ejection fracture
ILD	Interstitial lung disease
IPAH	Idiopathic pulmonary arterial hypertension
Ipc	Isolated postcapillary
JVD	Jugular venous distension
Kv	Potassium gated ion channel
LAP	Left-atrial pressure
LHC	Left-heart catheterization
LHD	Left-heart disease
LV	Left ventricle
LVEDP	Left-ventricular end-diastolic pressure
mPAP	Mean pulmonary artery pressure
NO	Nitric oxide
NT-pro BNP	N-terminal pro-B-type natriuretic peptide
OSA	Obstructive sleep apnea
PA	Pulmonary artery
PCA	Prostacyclin analog/agonist
PCWP	Pulmonary capillary wedge pressure
PDE-5	Phosphodiesterase 5
PDGF	Platelet-derived growth factor
PGI2	Prostaglandin I2
PLCH	Pulmonary Langerhans cell histiocytosis
PTE	Pulmonary thromboendoarterectomy
PVR	Pulmonary vascular resistance
RCT	Randomized control trial
RV	Right ventricle
SAPH	Sarcoidosis-associated PH
SCD	Scleroderma
SQ	Subcutaneous
TGF-B	Transforming growth factor-beta
TTCW	Time to clinical worsening
TTE	Transthoracic echocardiography
V/Q	Ventilation and perfusion
WHO	World Health Organization

## References

[1] Ruopp N.F., Farber H.W. (2019). The New World Symposium on Pulmonary Hypertension Guidelines: Should Twenty-One Be the New Twenty-Five?. Circulation.

[2] Naeije R., Richter M.J., Rubin L.J. (2022). The Physiological Basis of Pulmonary Arterial Hypertension. Eur. Respir. J..

[3] Hsu S. (2019). Coupling Right Ventricular-Pulmonary Arterial Research to the Pulmonary Hypertension Patient Bedside. Circ. Heart Fail..

[4] Brown L.M., Chen H., Halpern S., Taichman D., McGoon M.D., Farber H.W., Frost A.E., Liou T.G., Turner M., Feldkircher K. (2011). Delay in Recognition of Pulmonary Arterial Hypertension: Factors Identified from the REVEAL Registry. Chest.

[5] Humbert M., Kovacs G., Hoeper M.M., Badagliacca R., Berger R.M.F., Brida M., Carlsen J., Coats A.J.S., Escribano-Subias P., Ferrari P. (2022). 2022 ESC/ERS Guidelines for the Diagnosis and Treatment of Pulmonary Hypertension: Developed by the Task Force for the Diagnosis and Treatment of Pulmonary Hypertension of the European Society of Cardiology (ESC) and the European Respiratory Society (ERS). Endorsed by the International Society for Heart and Lung Transplantation (ISHLT) and the European Reference Network on Rare Respiratory Diseases (ERN-LUNG). Eur. Heart J..

[6] Rosenkranz S., Howard L.S., Gomberg-Maitland M., Hoeper M.M. (2020). Systemic Consequences of Pulmonary Hypertension and Right-Sided Heart Failure. Circulation.

[7] Tuder R.M. (2017). Pulmonary Vascular Remodeling in Pulmonary Hypertension. Cell Tissue Res..

[8] Hemnes A.R., Humbert M. (2017). Pathobiology of Pulmonary Arterial Hypertension: Understanding the Roads Less Travelled. Eur. Respir. Rev..

[9] Dieffenbach P.B., Maracle M., Tschumperlin D.J., Fredenburgh L.E. (2018). Mechanobiological Feedback in Pulmonary Vascular Disease. Front. Physiol..

[10] Sanz J., Kariisa M., Dellegrottaglie S., Prat-González S., Garcia M.J., Fuster V., Rajagopalan S. (2009). Evaluation of Pulmonary Artery Stiffness in Pulmonary Hypertension with Cardiac Magnetic Resonance. JACC Cardiovasc. Imaging.

[11] Domingo E., Grignola J.C., Aguilar R., Messeguer M.L., Roman A. (2017). Pulmonary Arterial Wall Disease in COPD and Interstitial Lung Diseases Candidates for Lung Transplantation. Respir. Res..

[12] Bertero T., Cottrill K.A., Lu Y., Haeger C.M., Dieffenbach P., Annis S., Hale A., Bhat B., Kaimal V., Zhang Y.-Y. (2015). Matrix Remodeling Promotes Pulmonary Hypertension through Feedback Mechanoactivation of the YAP/TAZ-MiR-130/301 Circuit. Cell Rep..

[13] Galiè N., Humbert M., Vachiery J.-L., Gibbs S., Lang I., Torbicki A., Simonneau G., Peacock A., Vonk Noordegraaf A., Beghetti M. (2015). 2015 ESC/ERS Guidelines for the Diagnosis and Treatment of Pulmonary Hypertension: The Joint Task Force for the Diagnosis and Treatment of Pulmonary Hypertension of the European Society of Cardiology (ESC) and the European Respiratory Society (ERS)Endorsed by: Association for European Paediatric and Congenital Cardiology (AEPC), International Society for Heart and Lung Transplantation (ISHLT). Eur. Respir. J..

[14] McLaughlin V.V., Shah S.J., Souza R., Humbert M. (2015). Management of Pulmonary Arterial Hypertension. J. Am. Coll. Cardiol..

[15] Simonneau G., Montani D., Celermajer D.S., Denton C.P., Gatzoulis M.A., Krowka M., Williams P.G., Souza R. (2019). Haemodynamic Definitions and Updated Clinical Classification of Pulmonary Hypertension. Eur. Respir. J..

[16] Douschan P., Kovacs G., Avian A., Foris V., Gruber F., Olschewski A., Olschewski H. (2018). Mild Elevation of Pulmonary Arterial Pressure as a Predictor of Mortality. Am. J. Respir. Crit. Care Med..

[17] Zolty R. (2020). Pulmonary Arterial Hypertension Specific Therapy: The Old and the New. Pharmacol. Ther..

[18] Thenappan T., Ormiston M.L., Ryan J.J., Archer S.L. (2018). Pulmonary Arterial Hypertension: Pathogenesis and Clinical Management. BMJ.

[19] Qaiser K.N., Tonelli A.R. (2021). Novel Treatment Pathways in Pulmonary Arterial Hypertension. Methodist Debakey Cardiovasc. J..

[20] Correale M., Ferraretti A., Monaco I., Grazioli D., Di Biase M., Brunetti N.D. (2018). Endothelin-Receptor Antagonists in the Management of Pulmonary Arterial Hypertension: Where Do We Stand?. Vasc. Health Risk Manag..

[21] Sommer N., Ghofrani H.A., Pak O., Bonnet S., Provencher S., Sitbon O., Rosenkranz S., Hoeper M.M., Kiely D.G. (2021). Current and Future Treatments of Pulmonary Arterial Hypertension. Br. J. Pharmacol..

[22] Galiè N., Manes A., Branzi A. (2003). Prostanoids for Pulmonary Arterial Hypertension. Am. J. Respir. Med..

[23] Mitchell J.A., Ahmetaj-Shala B., Kirkby N.S., Wright W.R., Mackenzie L.S., Reed D.M., Mohamed N. (2014). Role of Prostacyclin in Pulmonary Hypertension. Glob. Cardiol. Sci. Pract..

[24] Gaine S., Sitbon O., Channick R.N., Chin K.M., Sauter R., Galiè N., Hoeper M.M., McLaughlin V.V., Preiss R., Rubin L.J. (2021). Relationship Between Time from Diagnosis and Morbidity/Mortality in Pulmonary Arterial Hypertension: Results from the Phase III GRIPHON Study. Chest.

[25] Highland K.B., Crawford R., Classi P., Morrison R., Doward L., Nelsen A.C., Castillo H., Mathai S.C., DuBrock H.M. (2021). Development of the Pulmonary Hypertension Functional Classification Self-Report: A Patient Version Adapted from the World Health Organization Functional Classification Measure. Health Qual. Life Outcomes.

[26] Benza R.L., Miller D.P., Barst R.J., Badesch D.B., Frost A.E., McGoon M.D. (2012). An Evaluation of Long-Term Survival from Time of Diagnosis in Pulmonary Arterial Hypertension from the REVEAL Registry. Chest.

[27] Klings E.S., Machado R.F., Barst R.J., Morris C.R., Mubarak K.K., Gordeuk V.R., Kato G.J., Ataga K.I., Gibbs J.S., Castro O. (2014). An Official American Thoracic Society Clinical Practice Guideline: Diagnosis, Risk Stratification, and Management of Pulmonary Hypertension of Sickle Cell Disease. Am. J. Respir. Crit. Care Med..

[28] Sitbon O., Humbert M., Jaïs X., Ioos V., Hamid A.M., Provencher S., Garcia G., Parent F., Hervé P., Simonneau G. (2005). Long-Term Response to Calcium Channel Blockers in Idiopathic Pulmonary Arterial Hypertension. Circulation.

[29] Rich S., Kaufmann E., Levy P.S. (1992). The Effect of High Doses of Calcium-Channel Blockers on Survival in Primary Pulmonary Hypertension. N. Engl. J. Med..

[30] Sadushi-Koliçi R., Skoro-Sajer N., Zimmer D., Bonderman D., Schemper M., Klepetko W., Glatz J., Jakowitsch J., Lang I.M. (2012). Long-Term Treatment, Tolerability, and Survival with Sub-Cutaneous Treprostinil for Severe Pulmonary Hypertension. J. Heart Lung Transplant..

[31] Jing Z.-C., Parikh K., Pulido T., Jerjes-Sanchez C., White R.J., Allen R., Torbicki A., Xu K.-F., Yehle D., Laliberte K. (2013). Efficacy and Safety of Oral Treprostinil Monotherapy for the Treatment of Pulmonary Arterial Hypertension: A Randomized, Controlled Trial. Circulation.

[32] White R.J., Jerjes-Sanchez C., Bohns Meyer G.M., Pulido T., Sepulveda P., Wang K.Y., Grünig E., Hiremath S., Yu Z., Gangcheng Z. (2020). Combination Therapy with Oral Treprostinil for Pulmonary Arterial Hypertension. A Double-Blind Placebo-Controlled Clinical Trial. Am. J. Respir. Crit. Care Med..

[33] Spikes L.A., Bajwa A.A., Burger C.D., Desai S.V., Eggert M.S., El-Kersh K.A., Fisher M.R., Johri S., Joly J.M., Mehta J. (2022). BREEZE: Open-label Clinical Study to Evaluate the Safety and Tolerability of Treprostinil Inhalation Powder as Tyvaso DPI^TM^ in Patients with Pulmonary Arterial Hypertension. Pulm. Circ..

[34] Liu H., Chen X., Li J., Su S., Ding T., Shi C., Jiang Y., Zhu Z. (2016). Efficacy and Safety of Pulmonary Arterial Hypertension-Specific Therapy in Pulmonary Arterial Hypertension: A Meta-Analysis of Randomized Controlled Trials. Chest.

[35] Torres F., Farber H., Ristic A., McLaughlin V., Adams J., Zhang J., Klassen P., Shanahan W., Grundy J., Hoffmann I. (2019). Efficacy and Safety of Ralinepag, a Novel Oral IP Agonist, in PAH Patients on Mono or Dual Background Therapy: Results from a Phase 2 Randomised, Parallel Group, Placebo-Controlled Trial. Eur. Respir. J..

[36] Galiè N., Olschewski H., Oudiz R.J., Torres F., Frost A., Ghofrani H.A., Badesch D.B., McGoon M.D., McLaughlin V.V., Roecker E.B. (2008). Ambrisentan for the Treatment of Pulmonary Arterial Hypertension: Results of the Ambrisentan in Pulmonary Arterial Hypertension, Randomized, Double-Blind, Placebo-Controlled, Multicenter, Efficacy (ARIES) Study 1 and 2. Circulation.

[37] Pulido T., Adzerikho I., Channick R.N., Delcroix M., Galiè N., Ghofrani H.-A., Jansa P., Jing Z.-C., Le Brun F.-O., Mehta S. (2013). Macitentan and Morbidity and Mortality in Pulmonary Arterial Hypertension. N. Engl. J. Med..

[38] Humbert M. (2004). Combination of Bosentan with Epoprostenol in Pulmonary Arterial Hypertension: BREATHE-2. Eur. Respir. J..

[39] Rivera-Lebron B.N., Risbano M.G. (2017). Ambrisentan: A Review of Its Use in Pulmonary Arterial Hypertension. Ther. Adv. Respir. Dis..

[40] Galiè N., Barberà J.A., Frost A.E., Ghofrani H.-A., Hoeper M.M., McLaughlin V.V., Peacock A.J., Simonneau G., Vachiery J.-L., Grünig E. (2015). Initial Use of Ambrisentan plus Tadalafil in Pulmonary Arterial Hypertension. N. Engl. J. Med..

[41] Ghofrani H.-A., D’Armini A.M., Grimminger F., Hoeper M.M., Jansa P., Kim N.H., Mayer E., Simonneau G., Wilkins M.R., Fritsch A. (2013). Riociguat for the Treatment of Chronic Thromboembolic Pulmonary Hypertension. N. Engl. J. Med..

[42] Rubin L.J., Badesch D.B., Fleming T.R., Galiè N., Simonneau G., Ghofrani H.A., Oakes M., Layton G., Serdarevic-Pehar M., McLaughlin V.V. (2011). Long-Term Treatment with Sildenafil Citrate in Pulmonary Arterial Hypertension. Chest.

[43] Macchia A., Marchioli R., Marfisi R., Scarano M., Levantesi G., Tavazzi L., Tognoni G. (2007). A Meta-Analysis of Trials of Pulmonary Hypertension: A Clinical Condition Looking for Drugs and Research Methodology. Am. Heart J..

[44] Hoeper M.M., Al-Hiti H., Benza R.L., Chang S.-A., Corris P.A., Gibbs J.S.R., Grünig E., Jansa P., Klinger J.R., Langleben D. (2021). Switching to Riociguat versus Maintenance Therapy with Phosphodiesterase-5 Inhibitors in Patients with Pulmonary Arterial Hypertension (REPLACE): A Multicentre, Open-Label, Randomised Controlled Trial. Lancet Respir. Med..

[45] McLaughlin V., Channick R.N., Ghofrani H.-A., Lemarié J.-C., Naeije R., Packer M., Souza R., Tapson V.F., Tolson J., Al Hiti H. (2015). Bosentan Added to Sildenafil Therapy in Patients with Pulmonary Arterial Hypertension. Eur. Respir. J..

[46] Chin K.M., Sitbon O., Doelberg M., Feldman J., Gibbs J.S.R., Grünig E., Hoeper M.M., Martin N., Mathai S.C., McLaughlin V.V. (2021). Three- Versus Two-Drug Therapy for Patients with Newly Diagnosed Pulmonary Arterial Hypertension. J. Am. Coll. Cardiol..

[47] Badesch D., Gibbs S., Gomberg-Maitland M., Humbert M., Mclaughlin V., Preston I., Souza R., Waxman A., De Oliveira Pena J., Barnes J. (2019). PULSAR: A Phase 2, Randomized, Double-Blind, Placebo-Controlled Study to Assess the Efficacy and Safety of Sotatercept (ACE-011) when Added to Standard of Care (SOC) for Treatment of Pulmonary Arterial Hypertension (PAH). Eur. Respir. J..

[48] Humbert M., McLaughlin V., Gibbs J.S.R., Gomberg-Maitland M., Hoeper M.M., Preston I.R., Souza R., Waxman A., Escribano Subias P., Feldman J. (2021). Sotatercept for the Treatment of Pulmonary Arterial Hypertension. N. Engl. J. Med..

[49] Spiekerkoetter E., Sung Y.K., Sudheendra D., Bill M., Aldred M.A., van de Veerdonk M.C., Vonk Noordegraaf A., Long-Boyle J., Dash R., Yang P.C. (2015). Low-Dose FK506 (Tacrolimus) in End-Stage Pulmonary Arterial Hypertension. Am. J. Respir. Crit. Care Med..

[50] Chen J., Cui X., Qian Z., Li Y., Kang K., Qu J., Li L., Gou D. (2016). Multi-Omics Analysis Reveals Regulators of the Response to PDGF-BB Treatment in Pulmonary Artery Smooth Muscle Cells. BMC Genom..

[51] Hoeper M.M., Barst R.J., Bourge R.C., Feldman J., Frost A.E., Galié N., Gómez-Sánchez M.A., Grimminger F., Grünig E., Hassoun P.M. (2013). Imatinib Mesylate as Add-on Therapy for Pulmonary Arterial Hypertension: Results of the Randomized IMPRES Study. Circulation.

[52] Frantz R.P., Benza R.L., Channick R.N., Chin K., Howard L.S., McLaughlin V.V., Sitbon O., Zamanian R.T., Hemnes A.R., Cravets M. (2021). TORREY, a Phase 2 Study to Evaluate the Efficacy and Safety of Inhaled Seralutinib for the Treatment of Pulmonary Arterial Hypertension. Pulm. Circ..

[53] Frantz R.P., Highland K.B., McConnell J.W., Burger C.D., Roscigno R.F., Cravets M., McCaffrey R., Zisman L.S., Howard L. (2021). A Phase 1b, Multi-Center, Randomized, Placebo-Controlled Trial of Inhaled Seralutinib in Subjects with WHO Group 1 Pulmonary Arterial Hypertension. TP82. TP082 Let It Be—Clinical Advances in Pulmonary Vascular Disease: Pah and beyond, May.

[54] Archer S.L., Roach R.C., Hackett P.H., Wagner P.D. (2016). Acquired Mitochondrial Abnormalities, Including Epigenetic Inhibition of Superoxide Dismutase 2, in Pulmonary Hypertension and Cancer: Therapeutic Implications. Hypoxia: Translation in Progress.

[55] Dean A., Nilsen M., Loughlin L., Salt I.P., MacLean M.R. (2016). Metformin Reverses Development of Pulmonary Hypertension via Aromatase Inhibition. Hypertension.

[56] Safdar Z., Cho E. (2021). Effect of Spironolactone Use in Pulmonary Arterial Hypertension—Analysis from Pivotal Trial Databases. Pulm. Circ..

[57] Lazarus H., Denning J., Kamau-Kelley W., Wring S., Palacios M., Humbert M. (2021). ELEVATE 2: A Multicenter Study of Rodatristat Ethyl in Patients with WHO Group 1 Pulmonary Arterial Hypertension (PAH). Eur. Respir. J..

[58] Lazarus H.M., Denning J., Wring S., Palacios M., Hoffman S., Crizer K., Kamau-Kelley W., Symonds W., Feldman J. (2022). A Trial Design to Maximize Knowledge of the Effects of Rodatristat Ethyl in the Treatment of Pulmonary Arterial Hypertension (ELEVATE 2). Pulm. Circ..

[59] Al-Omary M.S., Sugito S., Boyle A.J., Sverdlov A.L., Collins N.J. (2020). Pulmonary Hypertension Due to Left Heart Disease: Diagnosis, Pathophysiology, and Therapy. Hypertension.

[60] Fang J.C., DeMarco T., Givertz M.M., Borlaug B.A., Lewis G.D., Rame J.E., Gomberg-Maitland M., Murali S., Frantz R.P., McGlothlin D. (2012). World Health Organization Pulmonary Hypertension Group 2: Pulmonary Hypertension due to Left Heart Disease in the Adult—A Summary Statement from the Pulmonary Hypertension Council of the International Society for Heart and Lung Transplantation. J. Heart Lung Transplant..

[61] Tedford R.J., Hassoun P.M., Mathai S.C., Girgis R.E., Russell S.D., Thiemann D.R., Cingolani O.H., Mudd J.O., Borlaug B.A., Redfield M.M. (2012). Pulmonary Capillary Wedge Pressure Augments Right Ventricular Pulsatile Loading. Circulation.

[62] Melenovsky V., Andersen M.J., Andress K., Reddy Y.N., Borlaug B.A. (2015). Lung Congestion in Chronic Heart Failure: Haemodynamic, Clinical, and Prognostic Implications: Congestion and Pulmonary Haemodynamics in HF. Eur. J. Heart Fail..

[63] Yancy C.W., Jessup M., Bozkurt B., Butler J., Casey D.E., Drazner M.H., Fonarow G.C., Geraci S.A., Horwich T., Januzzi J.L. (2013). 2013 ACCF/AHA Guideline for the Management of Heart Failure: A Report of the American College of Cardiology Foundation/American Heart Association Task Force on Practice Guidelines. Circulation.

[64] Califf R.M., Adams K.F., McKenna W.J., Gheorghiade M., Uretsky B.F., McNulty S.E., Darius H., Schulman K., Zannad F., Handberg-Thurmond E. (1997). A Randomized Controlled Trial of Epoprostenol Therapy for Severe Congestive Heart Failure: The Flolan International Randomized Survival Trial (FIRST). Am. Heart J..

[65] Anand I., McMurray J., Cohn J.N., Konstam M.A., Notter T., Quitzau K., Ruschitzka F., Lüscher T.F. (2004). Long-Term Effects of Darusentan on Left-Ventricular Remodelling and Clinical Outcomes in the Endothelin A Receptor Antagonist Trial in Heart Failure (EARTH): Randomised, Double-Blind, Placebo-Controlled Trial. Lancet.

[66] Vachiéry J.-L., Delcroix M., Al-Hiti H., Efficace M., Hutyra M., Lack G., Papadakis K., Rubin L.J. (2018). Macitentan in Pulmonary Hypertension Due to Left Ventricular Dysfunction. Eur. Respir. J..

[67] Kramer T., Dumitrescu D., Gerhardt F., Orlova K., ten Freyhaus H., Hellmich M., Baldus S., Rosenkranz S. (2019). Therapeutic Potential of Phosphodiesterase Type 5 Inhibitors in Heart Failure with Preserved Ejection Fraction and Combined Post- and Pre-Capillary Pulmonary Hypertension. Int. J. Cardiol..

[68] Ghofrani H.A., Grimminger F. (2009). Soluble Guanylate Cyclase Stimulation: An Emerging Option in Pulmonary Hypertension Therapy. Eur. Respir. Rev..

[69] Bonderman D., Ghio S., Felix S.B., Ghofrani H.-A., Michelakis E., Mitrovic V., Oudiz R.J., Boateng F., Scalise A.-V., Roessig L. (2013). Riociguat for Patients with Pulmonary Hypertension Caused by Systolic Left Ventricular Dysfunction: A Phase IIb Double-Blind, Randomized, Placebo-Controlled, Dose-Ranging Hemodynamic Study. Circulation.

[70] Gheorghiade M., Greene S.J., Butler J., Filippatos G., Lam C.S.P., Maggioni A.P., Ponikowski P., Shah S.J., Solomon S.D., Kraigher-Krainer E. (2015). Effect of Vericiguat, a Soluble Guanylate Cyclase Stimulator, on Natriuretic Peptide Levels in Patients with Worsening Chronic Heart Failure and Reduced Ejection Fraction: The Socrates-Reduced Randomized Trial. JAMA.

[71] Brittain E.L., Niswender K., Agrawal V., Chen X., Fan R., Pugh M.E., Rice T.W., Robbins I.M., Song H., Thompson C. (2020). Mechanistic Phase II Clinical Trial of Metformin in Pulmonary Arterial Hypertension. JAHA.

[72] McGettrick M., Peacock A. (2020). Group 3 Pulmonary Hypertension: Challenges and Opportunities. Glob. Cardiol. Sci. Pract..

[73] Singh N., Dorfmüller P., Shlobin O.A., Ventetuolo C.E. (2022). Group 3 Pulmonary Hypertension: From Bench to Bedside. Circ. Res..

[74] Chaouat A., Naeije R., Weitzenblum E. (2008). Pulmonary Hypertension in COPD. Eur. Respir. J..

[75] Minai O.A., Ricaurte B., Kaw R., Hammel J., Mansour M., McCarthy K., Golish J.A., Stoller J.K. (2009). Frequency and Impact of Pulmonary Hypertension in Patients with Obstructive Sleep Apnea Syndrome. Am. J. Cardiol..

[76] King C.S., Shlobin O.A. (2020). The Trouble with Group 3 Pulmonary Hypertension in Interstitial Lung Disease: Dilemmas in Diagnosis and the Conundrum of Treatment. Chest.

[77] King T.E., Brown K.K., Raghu G., du Bois R.M., Lynch D.A., Martinez F., Valeyre D., Leconte I., Morganti A., Roux S. (2011). BUILD-3: A Randomized, Controlled Trial of Bosentan in Idiopathic Pulmonary Fibrosis. Am. J. Respir. Crit. Care Med..

[78] King T.E., Behr J., Brown K.K., du Bois R.M., Lancaster L., de Andrade J.A., Stähler G., Leconte I., Roux S., Raghu G. (2008). BUILD-1: A Randomized Placebo-Controlled Trial of Bosentan in Idiopathic Pulmonary Fibrosis. Am. J. Respir. Crit. Care Med..

[79] Corte T.J., Keir G.J., Dimopoulos K., Howard L., Corris P.A., Parfitt L., Foley C., Yanez-Lopez M., Babalis D., Marino P. (2014). Bosentan in Pulmonary Hypertension Associated with Fibrotic Idiopathic Interstitial Pneumonia. Am. J. Respir. Crit. Care Med..

[80] Raghu G., Million-Rousseau R., Morganti A., Perchenet L., Behr J., MUSIC Study Group (2013). Macitentan for the Treatment of Idiopathic Pulmonary Fibrosis: The Randomised Controlled MUSIC Trial. Eur. Respir. J..

[81] Raghu G., Behr J., Brown K.K., Egan J.J., Kawut S.M., Flaherty K.R., Martinez F.J., Nathan S.D., Wells A.U., Collard H.R. (2013). Treatment of Idiopathic Pulmonary Fibrosis with Ambrisentan: A Parallel, Randomized Trial. Ann. Intern. Med..

[82] Zisman D.A., Schwarz M., Anstrom K.J. (2010). A Controlled Trial of Sildenafil in Advanced Idiopathic Pulmonary Fibrosis. N. Engl. J. Med..

[83] Nathan S.D., Behr J., Collard H.R., Cottin V., Hoeper M.M., Martinez F.J., Corte T.J., Keogh A.M., Leuchte H., Mogulkoc N. (2019). Riociguat for Idiopathic Interstitial Pneumonia-Associated Pulmonary Hypertension (RISE-IIP): A Randomised, Placebo-Controlled Phase 2b Study. Lancet Respir. Med..

[84] Waxman A., Restrepo-Jaramillo R., Thenappan T., Ravichandran A., Engel P., Bajwa A., Allen R., Feldman J., Argula R., Smith P. (2021). Inhaled Treprostinil in Pulmonary Hypertension Due to Interstitial Lung Disease. N. Engl. J. Med..

[85] Tian Z., Jiang X., Jing Z.-C. (2021). How Should a Physician Approach the Pharmacological Management of Chronic Thromboembolic Pulmonary Hypertension?. Expert Opin. Pharmacother..

[86] Kim N.H., Delcroix M., Jais X., Madani M.M., Matsubara H., Mayer E., Ogo T., Tapson V.F., Ghofrani H.-A., Jenkins D.P. (2019). Chronic Thromboembolic Pulmonary Hypertension. Eur. Respir. J..

[87] Simonneau G., Torbicki A., Dorfmüller P., Kim N. (2017). The Pathophysiology of Chronic Thromboembolic Pulmonary Hypertension. Eur. Respir. Rev..

[88] Jaïs X., D’Armini A.M., Jansa P., Torbicki A., Delcroix M., Ghofrani H.A., Hoeper M.M., Lang I.M., Mayer E., Pepke-Zaba J. (2008). Bosentan for Treatment of Inoperable Chronic Thromboembolic Pulmonary Hypertension: BENEFiT (Bosentan Effects in INopErable Forms of ChronIc Thromboembolic Pulmonary Hypertension), a Randomized, Placebo-Controlled Trial. J. Am. Coll. Cardiol..

[89] Escribano-Subias P., Bendjenana H., Curtis P.S., Lang I., Noordegraaf A.V. (2019). Ambrisentan for Treatment of Inoperable Chronic Thromboembolic Pulmonary Hypertension (CTEPH). Pulm. Circ..

[90] Ghofrani H.-A., Simonneau G., D’Armini A.M., Fedullo P., Howard L.S., Jaïs X., Jenkins D.P., Jing Z.-C., Madani M.M., Martin N. (2017). Macitentan for the Treatment of Inoperable Chronic Thromboembolic Pulmonary Hypertension (MERIT-1): Results from the Multicentre, Phase 2, Randomised, Double-Blind, Placebo-Controlled Study. Lancet Respir. Med..

[91] Sadushi-Kolici R., Jansa P., Kopec G., Torbicki A., Skoro-Sajer N., Campean I.-A., Halank M., Simkova I., Karlocai K., Steringer-Mascherbauer R. (2019). Subcutaneous Treprostinil for the Treatment of Severe Non-Operable Chronic Thromboembolic Pulmonary Hypertension (CTREPH): A Double-Blind, Phase 3, Randomised Controlled Trial. Lancet Respir. Med..

[92] Anthi A., Tsangaris I., Hamodraka E.S., Lekakis J., Armaganidis A., Orfanos S.E. (2012). Treatment with Bosentan in a Patient with Thalassemia Intermedia and Pulmonary Arterial Hypertension. Blood.

[93] Morris C.R., Kim H.-Y., Wood J., Porter J.B., Klings E.S., Trachtenberg F.L., Sweeters N., Olivieri N.F., Kwiatkowski J.L., Virzi L. (2013). Sildenafil Therapy in Thalassemia Patients with Doppler-Defined Risk of Pulmonary Hypertension. Haematologica.

[94] Kalantari S., Gomberg-Maitland M. (2016). Group 5 Pulmonary Hypertension: The Orphan’s Orphan Disease. Cardiol. Clin..

